# Spatial and Temporal Dynamics of Birch-Mining *Eriocrania* Moths in an Urban Landscape over Four Decades

**DOI:** 10.3390/insects17010005

**Published:** 2025-12-19

**Authors:** Mikhail V. Kozlov, Alexandr A. Egorov, Elena Valdés-Correcher, Vitali Zverev

**Affiliations:** 1 Department of Biology, University of Turku, 20014 Turku, Finland; 2 Laboratory of Wetland Studies, Institute of Forest Science, Russian Academy of Sciences, Uspenskoe, Moscow 143030, Russia; egorovfta@yandex.ru; 3 Center for Research on Desertification (CIDE), 46113 Moncada, Spain

**Keywords:** *Betula pendula*, *Betula pubescens*, distribution patterns, *Eriocrania*, St. Petersburg, urbanisation, extinction, colonization

## Abstract

Understanding how urbanisation shapes species distributions and ecological interactions requires long-term, spatially structured data. Using an exceptionally rare 40-year dataset (1986–2025) from 150 habitat patches and 102 downtown grid cells in St. Petersburg, Russia, we examined patterns in birch (*Betula pendula* and *B. pubescens*) persistence, ground conditions, woody vegetation, and the occurrence of *Eriocrania* leaf-mining moths. Birch presence, birch abundance, and ground quality declined both toward the city centre and over time, whereas woody plant cover showed no clear spatial or temporal pattern. *Eriocrania* occurrence within birch-containing patches was influenced primarily by habitat type, artificial ground, and birch abundance, while distance to the city centre, year, and woody cover exerted no consistent effects. Habitat characteristics offered only moderate predictive power for local extinction risk in both birches and *Eriocrania*, indicating that multiple drivers interact to shape patch dynamics. Contrary to the widespread declines observed in many insect taxa, *Eriocrania* populations exhibited no directional density trend across four decades. This long-term stability highlights the resilience of specialised herbivores in heterogeneous urban landscapes and underscores the value of extended temporal datasets for detecting subtle or unexpected ecological responses to urbanisation.

## Simple Summary

We studied how urbanisation affects birch trees and their moth herbivores in St. Petersburg, the second largest city in Russia, over four decades. While birch presence and patch quality declined near the city centre, moth populations remained stable, showing no long-term decline. Habitat characteristics partly predict local risks for birch and moths, but do not fully determine their survival in urban areas.

## 1. Introduction

The global expansion of urban areas reduces the extent of natural habitats, causing increasing fragmentation or replacement of natural areas by built environments. These changes affect both the availability and quality of habitats suitable for specific species [1,2,3,4]. As a result, some native species may become extinct from urban habitats due to heat or pollution stress or as a consequence of habitat management [5,6,7]. Others expand their ranges into cities, responding to warmer microclimates [8,9] or tracking host plants absent from surrounding natural areas [10]. Thus, urbanisation reshapes the distribution of plant and animal species across various scales, producing significant biogeographical effects—hosting isolated populations of some species while creating gaps in the occurrence of others [11,12].

In an urban landscape, habitat patches suitable for herbivorous insects are embedded within a matrix of buildings, roads and paved surfaces, covering up to 97% of the total area in downtown St. Petersburg [13]. Knowledge of the spatial arrangement of these patches and the temporal dynamics of their occupancy by particular species is critical for understanding patterns of urban biodiversity and identifying the drivers behind them [14]. However, long-term biogeographical studies in urban environments remain exceptionally rare. Most observational and experimental studies on within-city species distributions are restricted to a single year [1,15], leaving the temporal dynamics of local extinction and recolonisation poorly documented and insufficiently understood [16].

These dynamic processes are especially important in the extremely small habitats in the urban matrix, such as enclosed courtyards—often only a few dozen square meters in size—found in historical city centres. The acute shortage of data on species occurrences in these habitat patches over multiple years hampers the application of metapopulation theory [17] to describe how populations of species persist in a network of habitat patches within cities. Local populations with mutually independent within-patch dynamics may go extinct in some patches but can be recolonised from others, with connectivity, patch size, and habitat quality determining overall persistence, abundance, composition and distribution of biota under various urban development scenarios [18]. This particularly concerns informal greenery—such as courtyard plantings and remnant natural patches—the extent and dynamics of which remain poorly documented despite growing appreciation of its importance for maintaining urban biodiversity [19,20]. This study, focussing on native birches and birch-mining *Eriocrania* moths, seeks to address these research gaps.

Long-term datasets spanning several decades are widely regarded as the most effective for identifying factors that shape insect population dynamics [21], yet many studies rely on much shorter time series [22,23] because extended monitoring records remain uncommon [24]. Against this backdrop, our periodic surveys of *Eriocrania* moths in St. Petersburg, Russia (1986–2025), represent an exceptionally rare dataset that combines nearly four decades of continuity with substantial spatial replication.

The earliest data on *Eriocrania* distribution in St. Petersburg, Russia were collected in 1986 to classify urban areas by habitat suitability for these moths [25]. A follow-up survey in 2000–2001 aimed to evaluate changes in distribution following urban expansion. Unexpectedly, comparison with the 1986 baseline revealed colonisation of habitat patches previously unoccupied by *Eriocrania*—including in densely built downtown areas—rather than a decline in moth populations [26]. However, drawing conclusions about the contraction of an urban distribution gap of *Eriocrania* from only two time points proved problematic, as the observed patterns may have reflected natural cycles in habitat occupancy rather than a long-term directional trend.

Patch occupancy dynamics—a central concept in metapopulation theory [17]—are closely tied to long-term fluctuations in species population density. For *Eriocrania*, densities may vary 10- to 100-fold between consecutive years [27,28,29], making detection of directional changes difficult without long-term datasets. To address this limitation, the study was expanded into a monitoring project, with annual surveys conducted from 2000 to 2012 and concluding surveys carried out in 2023–2025. In the final stage of the project, data on woody plant cover were collected from space photographs taken in 1984 and 2023. The overarching goal was to improve our understanding of how urbanisation drives gradual, cumulative changes in species distributions and ecological interactions.

Using data uniformly collected from 1986 to 2025 on habitat features, birch occupancy and abundance, and the occurrence and population density of *Eriocrania* leafminers, we tested the following hypotheses: (1) The quality of habitat patches suitable for birches and birch-feeding *Eriocrania*—quantified by the presence of undisturbed natural soil, artificial ground (asphalt, sand and gravel), and the woody plant (tree and shrub) cover—declines with proximity to the city centre and over time, reflecting habitat degradation with increasing urbanisation. (2) Both the proportion of birch-occupied patches and the number of birch trees per patch decline towards the city centre and over time. (3) The occurrence and density of *Eriocrania* vary among habitat patches containing birches, and their spatial and temporal variations can be predicted from patch characteristics. (4) The occurrence and population density of *Eriocrania* in birch-containing patches decline with proximity to the city centre and over time. In this way, we aimed to improve the current understanding of how progressing urbanisation influences the fine-scale biogeography of specialised insect herbivores and to identify the conditions that enable these insects to persist or collapse within urban environments.

## 2. Materials and Methods

### 2.1. Study Area

St. Petersburg, known as Leningrad from 1924 to 1991 (coordinates of city centre, the Palace Square: 59°56′ N, 30°19′ E), is the second largest city in Russia. It was established in 1703 A.D. on a previously uninhabited terrain covered by mires and boreal swamp forests. The study region exhibits a cool, humid continental climate with maritime influences. The mean temperature is −4.8 °C in January and 19.1 °C in July, and annual precipitation averages 660 mm. The frost-free period lasts approximately five months, while the summer season spans three to three and a half months. The mean annual temperature in St. Petersburg over the past decades was approximately 1.2 °C higher than in surrounding areas [30] and increased from 5.7 °C to 6.7 °C between 1985 and 2024 [31]. Despite this warming, birch leafing in 2018–2021 occurred in the same dates as in 1980–2009 [32].

In the 1980s, the city’s administrative boundaries encompassed approximately 606 km^2^, confining the urban footprint largely to its historical core and Soviet-era residential districts [33]. From the mid-1980s to 2025 (the period covered by our study), St. Petersburg has experienced significant transformation, characterised by spatial expansion, demographic shifts, and evolving green infrastructure. New residential areas have emerged, expanding the city’s area to 1439 km^2^ (https://en.wikipedia.org/wiki/Saint_Petersburg; accessed on 19 December 2025). St. Petersburg’s official population has grown from approximately 4.9 million in 1986 to over 5.6 million by 2025 (https://www.macrotrends.net/global-metrics/cities/22365/saint-petersburg/population; accessed on 19 May 2025). However, considering unregistered residents and labour migrants, the actual population was estimated to already have reached 7 million in 2019 (https://www.sobaka.ru/city/society/138586; accessed on 19 May 2025).

During the late Soviet period, green infrastructure was centrally planned, resulting in numerous parks, public gardens and tree-lined streets. In 1986, when the study began, St. Petersburg maintained 14,922 hectares of green spaces, of which 4646 hectares were public [34]. By the end of 1996, these values had increased to 18,569 and 5999 hectares, respectively [35]. However, by 2022, public green spaces in the same area had shrunk to 3878 hectares (https://iac.spb.ru/upload/medialibrary/023/0237165c7c33682451724a3a90588f3f.pdf; accessed on 19 December 2025), marking a reversal in the urban greenery development. Along with this reduction in area, the quality of green spaces in St. Petersburg suffered from fragmentation, pollution and overuse [36].

### 2.2. Study System

White birches (*Betula pendula* Roth and *B. pubescens* Ehrhart) are deciduous, fast-growing, early successional broadleaved trees widespread across northern Eurasia. These iconic components of boreal forests, known for their distinctive white bark, serve as hosts to many herbivores, including eriocraniid moths—primarily *Eriocrania semipurpurella* (Stephens) sensu lato and *E. sangii* (Wood)—in northwestern Russia. The larvae of these small, metallic-coloured insects feed inside young birch leaves, forming large blotch mines (Figure S1) that often lead to premature leaf abscission. These species can reach high population densities in certain years, both in natural forests [29,37] and urban plantings [38], where they serve as a food source for birds and ants and support a diverse community of parasitic wasps [28]. However, *E. semipurpurella* and *E. sangii* cannot be reliably distinguished on the basis of their vacated mines (https://norfolkmoths.co.uk/micros.php; accessed on 4 December 2025).

*Eriocrania* prepupae and pupae spend nearly 11 months in the soil beneath their host trees [39]; thus, soil quality and the absence of physical disturbance are critical for their survival. This plant–herbivore system provides a valuable model for ecological and environmental studies [27,28,40].

### 2.3. Study Sites

The present study combines two data collection approaches that can conventionally be described as ecological and distributional. The first approach focused on understanding ecological processes and their drivers by selecting habitat patches replicated across space and studying the organisms within them. The second approach aimed to reveal spatial patterns across the urban landscape by dividing a representative part of it into a grid of uniform sampling units (cells hereafter), with no a priori knowledge of the occurrence of study objects in these cells, and surveying the selected groups of biota across this grid. These approaches are complementary, as the ecological method emphasises mechanisms, while the distributional method captures patterns. Their simultaneous use was intended to advance an understanding of how urban environments shape the occurrence of host plants (birches) and their specialist herbivores (*Eriocrania*) at low spatial scales.

**Figure 1:**
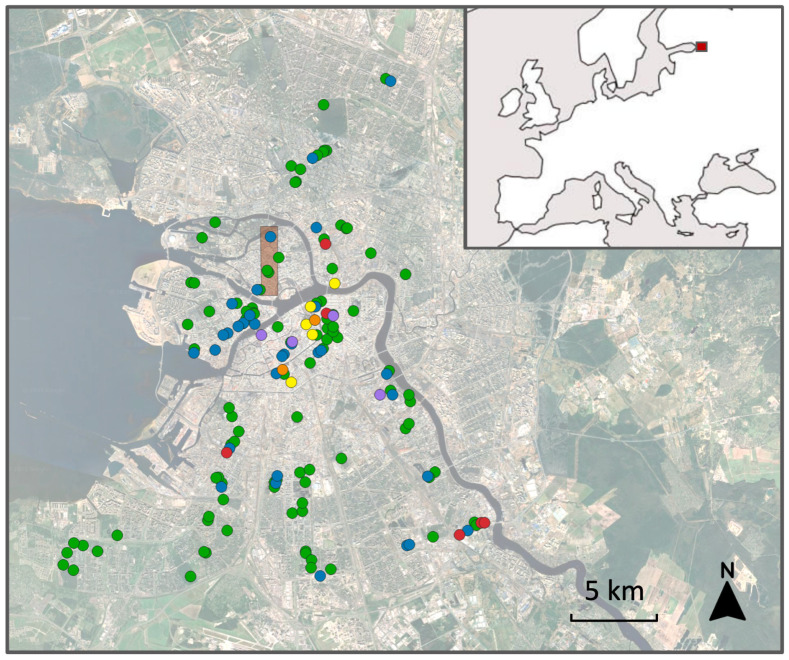
Position of 150 habitat patches (circles) within the city of St. Petersburg and changes in their type and birch presence between 1986 and 2025. Legend: blue, patches where birches occurred in 1986 but went extinct later; green, patches where birches persisted from 1986 to 2025; orange, patches where birches were absent in 1986 but planted later; purple, patches inaccessible in 2025; red, irreversibly destroyed patches; yellow, patches where birches were absent throughout the entire observation period. Brown rectangle: area divided by 105 grid cells. Inset: location of study area (red square) in Europe.

An ecological approach was implemented by haphazardly selecting 173 habitat patches in 1986 to evenly represent varying levels of urbanisation (as quantified primarily by the extent of impervious cover) and different habitat types, including public parks, public gardens, roadsides, open courtyards, enclosed courtyards, and wastelands (Figure S2). In 2000, we excluded 16 patches whose exact locations could not be reliably determined and merged patches situated 15–30 m apart. As a result, data were subsequently collected from 150 patches (Figure 1; Data S1). Seven of these patches were selected in historical public gardens that lacked birches in 1986 to assess whether birches would be planted later; the remaining 143 patches each contained one to more than 25 birches at that time.

A distributional approach was originally applied across four islands in the Neva River—Aptekarsky, Artillerysky, Petrogradsky and Zayachy—covering a total area of 8.75 km^2^. Presence/absence data for birch and *Eriocrania* were collected using a regular 160 × 160 m grid. In 1986, 379 grid cells, which covered an entire area of these islands, were surveyed. For feasibility reasons, surveys conducted in 2000, 2001, 2006–2012 and 2025 were each limited to 102 or fewer cells (Figure 1; Data S2), with the northeastern corner of the study area located at 59°58′43″ N, 30°18′39″ E, and the southwestern corner located at 59°56′58″ N, 30°17′38″ E (Figure 1).

Following an approach widely used in urban ecology [41,42,43], we used distance from the city centre as a proxy for the urbanisation gradient. Specifically, we measured, in Google Earth, the distance from each patch and grid cell to the urban centre of St. Petersburg (Palace Square). Distance from the urban core is correlated with multiple environmental factors associated with urbanization [44] and, for St. Petersburg in particular, has been shown to relate to variation in vegetation cover and to characteristics of plant and insect populations [2,13].

### 2.4. Ground Surveys

All habitat patches—with a few exceptions due to logistical constraints—were surveyed from June to early July 1986 (by M.V.K.) and in 2000, 2012 (by M.V.K. and V.Z. in both years) and 2025 (mainly by A.A.E., with several patches assessed by M.V.K. and V.Z.). During each survey, we recorded all ground types present under birch canopies or, if birches were absent, at a representative site within the patch. The following ground types were distinguished: three natural or semi-natural types—undisturbed, loosened (e.g., by digging) and trampled (i.e., compacted by foot traffic)—and three artificial—asphalt, sand and gravel. In patches with clearly defined borders (e.g., enclosed courtyards), we counted all birch individuals, from juveniles over 50 cm tall to mature trees, whereas in patches with indefinite borders (e.g., in public parks), we categorised the size of surveyed birch tree groups as small (1–3 trees), medium (4–10 trees) or large (>10 trees). We also estimated the heights of the birch trees (minimal to maximal, if more than one birch was present in a patch), birch species (in 2025 only) and the density of *Eriocrania* mines. In some sites, mine density (but not patch features) was also recorded in 1990 (by M.V.K.), 2001–2011 (by M.V.K. and V.Z. in all years), 2023 (by M.V.K.) and 2024 (by M.V.K. and A.A.E.).

In the lower parts of birch crowns (up to 5 m above the ground), mines were searched for visually, bending down high branches whenever possible. If no mines were found there, the upper parts of birch crowns were examined using 8× binoculars (until 2012) or 25× optical magnification with a Canon PowerShot SX620 HS camera, Tokyo, Japan (in 2025). The *Eriocrania* population density was classified as ‘low’ if it took more than 1 min to find the first mine. If the first mine was found within 1 min, the search continued. If a second mine was also found within the next minute, the density was classified as ‘high’; otherwise, it remained ‘low’. The conclusion ‘no mines found’ was based on the following search durations: one large birch tree (10+ m high): 2–3 min; group of 2–5 birches: 5 min; group of 6–15 birches: 8–10 min; group of 16+ birches: 12–15 min. If a detailed inspection of the birch crown was not possible—due to restricted site access, for example—the data on leafminer occurrence were considered missing.

Data collection in grid cells followed a different protocol. We differentiated between cells ‘not visited’ in a given year and cells containing only birches that were ‘not accessed’ to check for the presence of mines. Cells without a single birch were marked as ‘unsuitable’ (for *Eriocrania*), while those containing birches were classified as ‘suitable’ and further categorised as ‘occupied’ if at least one *Eriocrania* mine was recorded or ‘vacant’ if no *Eriocrania* mines were found.

### 2.5. Remote Sensing

We used high-resolution satellite imagery for 1984 and 2023 to quantify the percentage of tree and shrub cover within a 30 m radius around habitat patch centres. For 1984, we complemented the satellite data with historical black-and-white aerial imagery obtained from the www.retromap.ru (accessed on 1 September 2025), with a ground resolution of ~1.2 m/pixel. This dataset allowed us to capture fine-scale vegetation patterns not discernible in coarser satellite products available for that period. For 2023, we relied on very-high-resolution imagery from Google Earth (© Maxar Technologies, Westminster, CO, USA), with a native panchromatic resolution of ~0.3 m/pixel.

All imagery was first orthorectified and georeferenced to WGS84/UTM using stable control points identifiable in both periods (e.g., road intersections, isolated buildings). Within each 30 m buffer, tree and shrub cover was mapped by manually digitizing polygons corresponding to woody vegetation. Because classification relied exclusively on visual interpretation, polygons were delineated only from homogeneous patches that were visually unambiguous in both periods. To ensure consistency, the same annotator (E.V.-C.) produced all polygons following an internally standardised protocol defining minimum mapping units and class criteria.

We defined woody vegetation as any ligneous plant (tree or shrub) with a closed canopy visible from nadir. Individual crowns or crown clusters were included if their visible canopy diameter exceeded 1 m, reflecting the minimum resolvable object size across datasets. In cases where pixels or small patches contained mixed vegetation (e.g., shrubs interspersed with herbaceous cover), classification followed a majority rule: the polygons were assigned to the woody-vegetation class only if at least 50% of their area corresponded to woody canopy. Pixels for which woody cover could not be reliably assessed in the 1984 imagery were excluded; consequently, analyses were restricted to the 92 patches for which woody-cover delineation was feasible in both periods.

To assess classification consistency, 15% of the polygons (randomly selected) were re-examined by the same observer who performed the original delineation. This qualitative check showed generally high consistency, with discrepancies primarily linked to shadowed areas and heterogeneous shrub–grass mixtures in the 1984 imagery.

### 2.6. Data Analysis

Presence–absence data on ground types (Hypothesis 1), birches (Hypothesis 2) and *Eriocrania* (Hypothesis 3) were analysed using Generalised Linear Mixed Models (GLMMs) implemented in the SAS 9.2 GLIMMIX procedure with type III sums of squares. A binomial error distribution with a logit link function was applied. For categorical data on birch group size (small, medium, large; Hypothesis 2) and *Eriocrania* density (zero, low, high; Hypothesis 3), a multinomial error distribution with a cumulative logit (cumlogit) link function was used. All models included year as a fixed effect and patch or cell identifier as a random effect. The significance of random effects was evaluated using likelihood ratio tests [45].

If GLMMs failed to converge or if the properties of the data did not allow their use, class-level differences were assessed using the non-parametric Kruskal–Wallis test (SAS NPAR1WAY procedure). Spatial and temporal trends in continuous variables (Hypotheses 1, 2 and 4) were examined using either linear or quadratic regression models (SAS REG procedure) or by Pearson correlation coefficients based on either original or standardised values of the study variables, or by point-biserial correlation (SAS CORR procedure). Associations between nominal variables (Hypotheses 1 to 4) were analysed with chi-square tests (SAS FREQ procedure). Differences in woody plant cover between 1984 and 2023 (Hypothesis 1) were compared using paired t-tests (SAS TTEST procedure [45]).

## 3. Results

### 3.1. Ground Types Occurrence in Patches

Undisturbed natural soil was present in 78–80% of surveyed patches, and its occurrence did not change across study years (GLMM, fixed effect: *F*_3, 418_ = 0.16, *p* = 0.93). This stability resulted from a balance between the disappearance and appearance of undisturbed soil, each recorded in 23 patches (Data S1). The presence of this soil type increased with greater distance from the city centre (point-biserial correlation: *r* = 0.21, *n* = 150 patches, *p* = 0.0091) and varied significantly among habitat types (Kruskal–Wallis test: χ^2_5_^ = 88.9, *p* < 0.0001), ranging from 47.9% in enclosed courtyards to 100% in parks.

In contrast, the percentage of patches containing artificial ground (asphalt, sand or gravel) increased steadily from 19% in 1986 to 53% in 2025 (*F*_3, 417_ = 17.7, *p* < 0.0001). This increase was driven by the appearance of artificial ground in 60 patches, outweighing its disappearance in only 15 patches (Data S1). The occurrence of artificial ground tended to decrease with increasing distance from the city centre (point-biserial correlation: *r* = –0.16, *n* = 150 patches, *p* = 0.057) and varied significantly among habitat types (Kruskal–Wallis test: *χ*^2_5_^ = 47.5, *p* < 0.0001), ranging from 8.6% in parks to 50.7% in enclosed courtyards.

### 3.2. Tree and Shrub Cover in Patches

Woody plant cover within a 30 m buffer ranged from 0% to 100% (Data S1). Between 1984 and 2023, woody plant cover decreased in 54 patches and increased in 38 patches, indicating no overall trend (paired test: *t*_91_ = 1.29, *p* = 0.20). Mean woody plant cover across 1984 and 2023 did not vary with distance from the city centre (*r* = 0.07, *n* = 92 patches, *p* = 0.51), but differed significantly among habitat types (Kruskal–Wallis test: *χ*^2_4_^ = 39.1, *p* < 0.0001), ranging from 11.6% in enclosed courtyards to 66.8% in parks.

### 3.3. Birch Occurrence in Patches

Six of the 150 habitat patches (Figure 1) became permanently unsuitable for birches during the observation period. One enclosed courtyard (#8 in Data S1) was covered with a glass roof during building renovation; one public garden (#86) and one wasteland (#132) were fully or nearly fully asphalted; and three roadside habitats (#77, #136 and #137) were subsumed by expanded or newly built roads.

**Figure 2:**
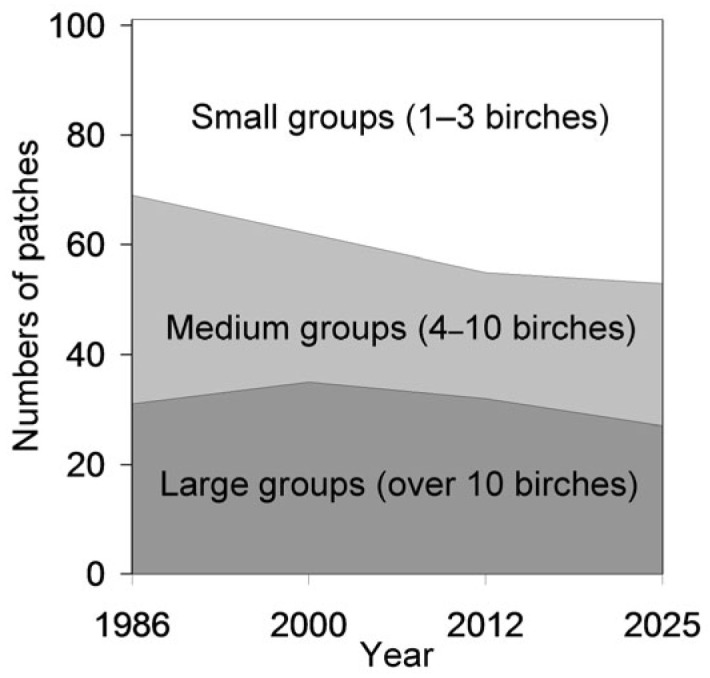
Distribution of 101 habitat patches in which birches persisted from 1986 to 2025 according to birch group size.

**Figure 3:**
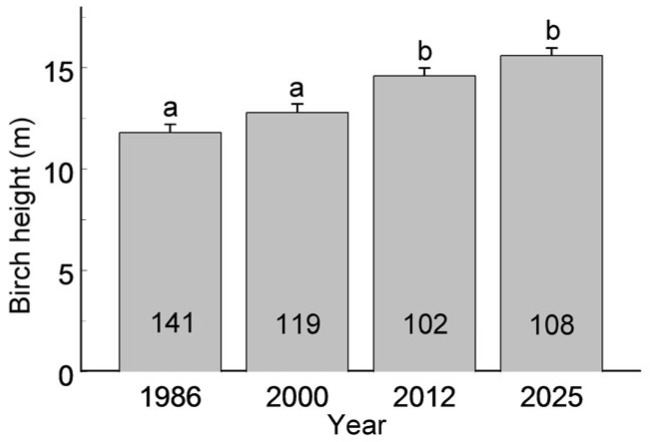
Height of birches (mean + S.E.) during the four main censuses. Sample sizes (numbers of habitat patches in which birches were measured in a given year) are shown within the bars. Bars marked with different letters differ significantly at *p* = 0.05.

The distribution of habitat patches by birch group size, categorised as small, medium or large, varied among years (*F*_3, 299_ = 3.90, *p* = 0.0094), with the proportion of small groups increasing from 31.7% in 1986 to 47.5% in 2025 (Figure 2). At the same time, the average birch height increased steadily over the study period (Figure 3; *F*_3, 466_ = 18.9, *p* < 0.0001).

The proportion of patches containing birches declined from 95.3% in 1986 to 73.4% in 2025 (*F*_3, 417_ = 11.1, *p* < 0.0001). This decrease was driven by birch disappearance from 36 patches, which outweighed their appearance in only four patches, two of which (#14 and #56 in Data S1) lacked birches in 1986. The probability of birch extinction over the observation period was two times higher in patches with artificial ground than in those without it and three times higher in patches with large birches (15–20 m tall in 1986) than in those with smaller trees. However, birch extinction was not associated with the presence of natural undisturbed soil (frequency analysis: *χ*^2_1_^ = 0.31, *p* = 0.58) or with the woody plant cover in 1986 (Kruskal–Wallis test: *χ*^2_1_^ = 0.96, *p* = 0.33).

Birch occurrence increased with distance from the city centre in both 1986 (*r* = 0.23, *n* = 150 patches, *p* = 0.004) and 2025 (*r* = 0.16, *n* = 150 patches, *p* = 0.061) and varied significantly among habitat types (Kruskal–Wallis test: χ^2_5_^ = 34.5, *p* < 0.0001), ranging from 60% in wastelands to 98.6% in parks.

Among patches in which birches were present in 2025, the majority (56.7%) contained *B. pendula*, 20.2% contained *B. pubescens*, and the remaining 23.1% harboured both species. Overall, *B. pendula* was twice as frequent in the study patches than was *B. pubescens* (Data S1), but its proportion in the birch population did not depend on the patch proximity to the city centre (*r* = 0.02, *n* = 104 patches, *p* = 0.81).

### 3.4. Birch Occurrence in Grid Cells

**Figure 4:**
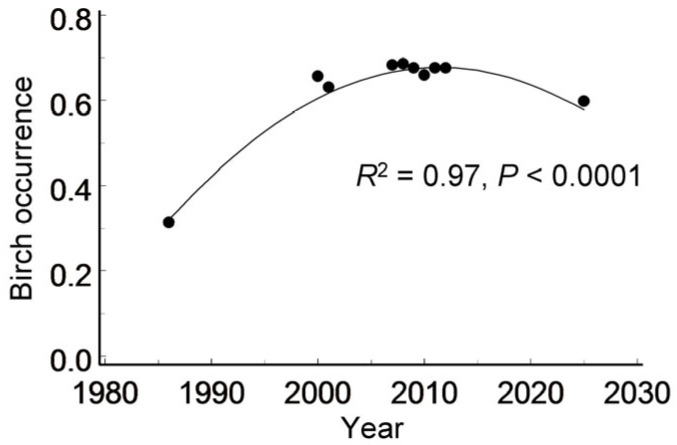
Variation in birch occurrence across 102 grid cells from 1986 to 2025. Line shows the quadratic regression.

The proportion of grid cells in which birches (of any size) were recorded (Data S2) varied both spatially (i.e., among individual cells: *χ*^2_1_^ = 197.7, *p* < 0.0001) and temporally (i.e., among years: *F*_9, 900_ = 8.48, *p* < 0.0001). This proportion approximately doubled between 1986 and 2000, remained relatively stable over the following decade and then declined slightly by 2025 (Figure 4). Spatially, birch occupancy decreased with increasing proximity to the city centre (point-biserial correlation: *r* = −0.27, *n* = 102 cells, *p* = 0.0060).

### 3.5. Eriocrania Occurrence in Patches

*Eriocrania* mines were recorded at least once in all 96 habitat patches where birches persisted throughout the observation period and where surveys were conducted in at least five years (Data S1). However, none of the 31 patches surveyed for 10 or more years showed uninterrupted *Eriocrania* occurrence. In these patches, we typically observed two or three cycles of local extinction, followed by recolonisation (Data S1).

**Figure 5:**
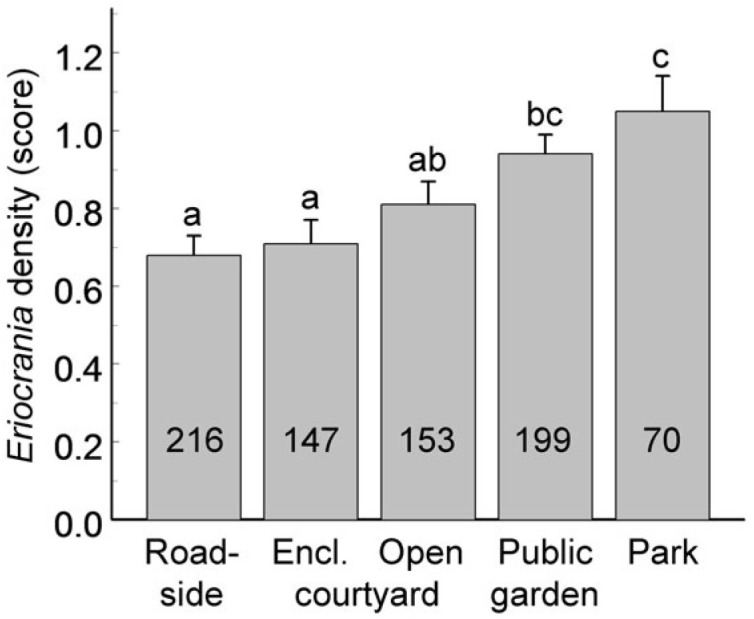
Variation in *Eriocrania* population density (mean ± S.E.) across habitat types over the entire observation period. Sample sizes (number of density assessments) are shown within the bars. Bars marked with different letters differ significantly at *p* = 0.05.

The *Eriocrania* population density, categorised as zero, low or high, varied significantly among habitat patches (GLMM, random effect: *χ*^2_1_^ = 19.8, *p* < 0.0001), and this variation was associated with the habitat type (Figure 5; F_4, 627_ = 18.2, *p* < 0.0001).

In 41 patches in which *Eriocrania* population density was quantified in at least five years, the average density increased with woody plant cover (*r* = 0.33, *n* = 41, *p* = 0.03); however, this correlation disappeared when the cover data were standardised by habitat type (*r* = 0.12, *n* = 41, *p* = 0.45). *Eriocrania* mines were recorded only in 33.0% of the patches lacking undisturbed soil in the observation year, whereas the occupancy of patches with undisturbed soil was much greater (65.4%; frequency analysis: *χ*^2_2_^ = 31.9, *p* < 0.0001). The occupancy of patches harbouring small- and medium-sized birch groups was 50–55%, but reached 73% for large-sized groups (*χ*^2_2_^ = 24.5, *p* < 0.0001).

**Figure 6:**
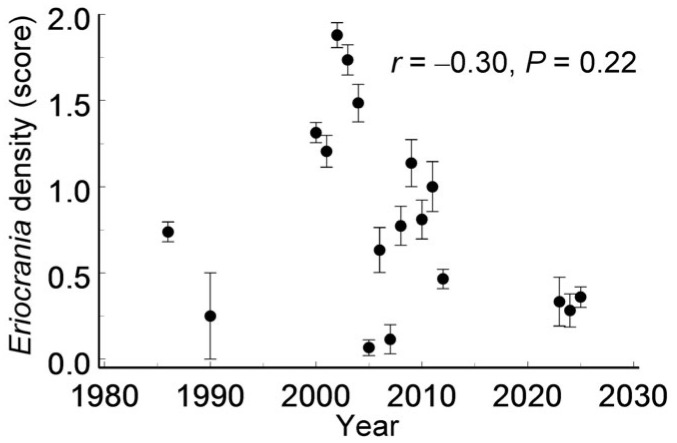
Among-year variation in *Eriocrania* population density (mean ± S.E.) across habitat patches from 1986 to 2025. Sample sizes: 1986—130; 1990—8; 2000—102; 2001—34; 2002—33; 2003—34; 2004—33; 2005, 2006—30; 2007—26; 2008, 2009—22; 2010—21; 2011—20; 2012—86; 2023—12; 2024—39; 2025—103.

Although the *Eriocrania* population density varied substantially across years (*F*_17, 627_ = 18.2, *p* < 0.0001), it showed no directional trend over time (Figure 6) and was unrelated to the distance from the city centre (*r* = 0.09, *n* = 140 patches, *p* = 0.27).

### 3.6. Eriocrania Occurrence in Grid Cells

*Eriocrania* mines were recorded at least once in 86 of 89 grid cells in which birches were observed at any year during the observation period (Data S2). However, only two of the 67 grid cells in which birches were recorded in at least five years exhibited an uninterrupted *Eriocrania* presence. Typically, grid cells showed two extinction events and two colonisation events over the study period (Data S2).

**Figure 7:**
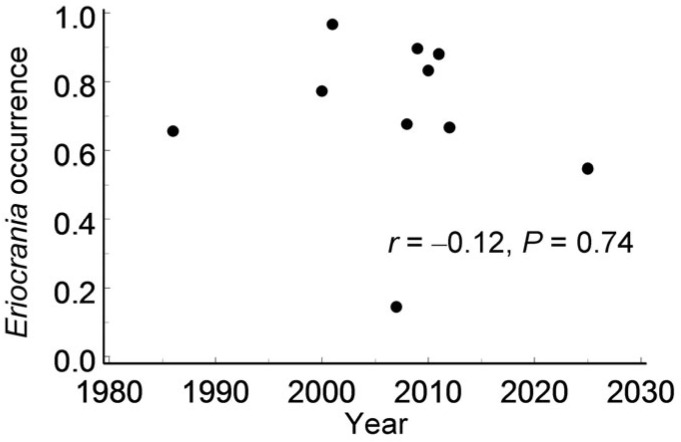
Variation in the proportion of grid cells with *Eriocrania* mines among grid cells populated by birches between 1986 and 2025.

The *Eriocrania* occurrence across grid cells (Data S2) varied significantly in both space (*χ*^2_1_^ = 16.6, *p* < 0.0001) and time (*F*_9, 509_ = 12.2, *p* < 0.0001). The annual proportion of grid cells occupied by *Eriocrania* ranged from 14.5% to 96.7%, but it showed no directional trend over time (Figure 7) or with distance from the city centre (*r* = 0.10, *n* = 89 cells, *p* = 0.34).

## 4. Discussion

### 4.1. Observed Patterns and Alignment with Hypotheses

Birch presence, abundance, and ground quality declined toward the city centre and over time, supporting Hypothesis 1, whereas woody plant cover showed no clear spatial or temporal trend, contrary to this hypothesis. Consistent with Hypothesis 2, the proportion of patches containing birches decreased over time but increased with distance from the city centre, resulting in higher occupancy in peripheral areas. Support for Hypothesis 3 was mixed: *Eriocrania* occurrence within birch-containing patches was influenced by habitat type, artificial ground, and birch abundance, but these factors only moderately predicted local extinction risk. Distance from the city centre and year had no consistent effect on *Eriocrania* occurrence, providing no support for Hypothesis 4.

### 4.2. Soil Quality

Our long-term study reveals that multiple interacting environmental factors influence the dynamics of the birch–*Eriocrania* interaction in urban landscapes. Among these, ground quality emerges as a potentially important—but often overlooked—component. Soil quality is especially critical for insects such as *Eriocrania*, as their larvae—in common with many insect species—burrow into the soil and construct protective chambers lined with silk impregnated with soil particles [46]. The larvae of *Eriocrania* remain in the soil for an extended period—around 11 months—before emerging as adults [39]. However, the relative importance of specific soil characteristics—such as type, texture, density, moisture and pH—for the survival of insects pupating in soil has only been explored in a limited number of species [46,47,48,49], and *Eriocrania* is not among them. Our observations on the relationship between the ground quality beneath birch canopies and the occurrence and density of *Eriocrania* mines in a patch provide preliminary insights into this issue.

Since *Eriocrania* larvae that drop from leaf mines cannot dig into asphalt or heavily compacted soil, they must crawl in search of a patch of undisturbed ground. These patches may lie several metres away from birch canopy projection, and the increased distance reduces larval survival due to desiccation and predation risk. This likely explains why *Eriocrania* mines were observed twice as frequently in habitat patches with natural undisturbed soil under birches compared to patches without it.

The occurrence of *Eriocrania* in patches lacking undisturbed soil may result from the migration of adults emerging in nearby habitat patches that offer a better environment for *Eriocrania*. As long as these migrants seem not to reliably discriminate between habitats with different soil suitability for safe overwintering, patches lacking undisturbed natural soil may act as death traps for the progenies of dispersing *Eriocrania* females. Alternatively, the occurrence of *Eriocrania* in these patches may indicate that larvae occasionally survive in seemingly unsuitable microsites, such as cracks in asphalt.

Soil quality for biota generally declines along urbanisation gradients due to disturbance (e.g., mowing, tilling), pollution, sealing and compaction [50,51,52], and our data from St. Petersburg are consistent with these patterns. Specifically, increases in soil sealing towards the city centre and from 1986 to 2025 support our Hypothesis 1, namely, that the soil quality for the birch–*Eriocrania* system declines both with proximity to the city centre and with the observation year, even though land management, in some patches, has resulted in the appearance of undisturbed soils where none previously existed.

Nonetheless, the modest decline in undisturbed soil occurrence does not appear to have had a fatal or severe impact on our study organisms, although it tended to increase the probability of birch extinction, in line with earlier studies that demonstrated adverse effects of soil sealing on tree vitality [53,54]. However, over a 40-year period, only six of 150 patches showed complete soil sealing that rendered habitats entirely unsuitable for birches and *Eriocrania*. Therefore, we conclude that while changes in soil quality are important, their influence is weaker on *Eriocrania* distribution and abundance in urban habitats than the effects of changes in birch populations.

### 4.3. Birch Population Dynamics

The proportion of birch-occupied patches and the number of birch trees per patch both declined with proximity to the city centre and over time. In contrast, woody plant cover showed no spatial or temporal changes. This discrepancy suggests that the availability of birches for specialised insect herbivores is shaped by factors distinct from those regulating overall urban greenery, namely, social and historical influences [55].

The tree species composition in the green areas of St. Petersburg has undergone significant changes over decades and centuries. During the 1950s–1960s, poplars (*Populus* spp.) were planted most intensively, and by the start of our study in the 1980s, they had become the most abundant tree species in the city [56]. In the 1970s–1980s, limes (*Tilia* spp.) accounted for 25% and maples (*Acer* spp.) for 17% of all seedlings cultivated in city nurseries, whereas birches (*Betula* spp.) comprised only about 5% [57]. However, by the mid-1990s, the proportion of birches among newly planted trees increased to 25–30% [26]. This trend is corroborated by our data showing a doubling in the proportion of grid cells that have a birch presence in the Petrogradsky district of St. Petersburg between 1986 and 2000 (Figure 4).

A subsequent decrease in the number of birch-occupied cells aligns with previous findings of a temporal shift in birch population trends in St. Petersburg—from an increase in 1986–2000 to a decline over the past two decades [13]. However, this decline did not reduce birch occurrence in the Petrogradsky district to the level observed in 1986. Contrary to our Hypothesis 2, grid-cell data indicate an overall increase in birch occurrence over the past 40 years (Figure 4). This conclusion may be spatially limited, as our observations revealed local extinctions in 23% of the patches in which birches had been present in 1986 (Figure 1). This trend is further reflected in the growing proportion of small birch groups (1–3 individuals), which increased from 32% in 1986 to 48% in 2025 (Figure 2). The concurrent decline in both the number and size of birch-occupied patches suggests an increase in population fragmentation, which may reduce connectivity among birch groups [57,58] and compromise dispersal corridors for birch-feeding insects.

An increase in birch height (Figure 3), which corresponds to a 50% rise in foliar biomass per average tree (based on equations provided in [59]), may currently offset the decline in birch abundance, thereby maintaining relatively stable food resources for *Eriocrania*. Specifically, in the historic city centre, smaller numbers of birches are offset by taller trees with higher foliar biomass, whereas in peripheral patches, birch numbers remain higher but biomass per tree shows more variable trends due to differences in recruitment and local management practices. Thus, our findings offer only partial support for Hypothesis 2. While birch occurrence declines towards the city centre, temporal trends are more nuanced and depend on the response variable (tree number vs. foliar biomass) and study approach (ecological vs. distributional). This discrepancy further challenges the reliability of using space-for-time substitution to predict temporal changes in ecosystem structure and functions [60], particularly in urban ecological contexts [13].

The continuous increase in average birch height across the city (Figure 3) points to a lack of recruitment—either via planting or natural regeneration—and supports earlier findings on the ageing of birch populations in St. Petersburg [13]. This trend suggests that the observed mismatch between spatial and temporal patterns in birch occurrence along the urbanisation gradient may soon be resolved through a rapid decline in birches in urban habitats. Birches typically begin to develop heartwood discolouration and rot at 60–70 years of age, which reduces their vitality and increases their vulnerability to windthrow [61]. Unless tree planting is substantially increased in the coming years—as outlined in the St. Petersburg government’s plan to achieve 30% green coverage of the metropolitan area by 2030 (https://gorod-plus.tv/news/141712; accessed on 19 December 2025)—a large-scale birch decline is likely, with significant implications for the composition of the urban tree community and the persistence of birch-associated biota.

### 4.4. Eriocrania Population Dynamics

The spatial population structure of any species depends on both the distribution of its habitat and the species’ ability to traverse the distances separating habitat patches [62,63]. Consistent with the classical metapopulation model [17], we observed a dynamic balance of colonisation and extinction events for *Eriocrania* among discrete habitat patches. Contrary to expectations rooted in the generally adverse effects of urbanisation on biota [64,65], none of the suitable patches—defined as those containing birches—remained persistently unoccupied by *Eriocrania* for over a decade, even when the patch was limited to a single birch tree in the historic city centre.

The birch population density per unit area (including impervious surfaces) in downtown St. Petersburg averages 14% of that in the uptown [13]. As a result, fine-scale dot maps based on single-year data [25,26] may suggest gaps in the distribution of both birches and *Eriocrania*. However, as the observation period extends, the number of patches that have ever been colonised increases, challenging these interpretations. Given that the nearest neighbour distance between birch-inhabited patches in the downtown area rarely exceeds 150 m (M.V.K., pers. obs.), and that *Eriocrania* occupancy is unrelated to proximity to the city centre, we conclude that the urban core does not represent a true lacuna in the *Eriocrania* distribution within the St. Petersburg metropolitan area.

Likewise, no patch—except for large public parks—was continuously occupied by *Eriocrania* throughout the study period. This suggests that local recruitment often fails to offset mortality and implies that *Eriocrania* populations in small habitat patches function as demographic sinks (sensu [66]). Thus, the key remaining questions are which populations act as demographic sources and how far they are from these sinks.

Most parks with substantial birch populations are located more than 5 km from the city centre, meaning that sink populations in the downtown areas are separated from potential sources by several kilometres of densely built terrain. The dispersal capacity of *Eriocrania* females is unknown, but that of the similarly sized leafminer *Tischeria ekebladella* (Bjerkander) does not exceed 100 m [63]. Thus, direct colonisation of vacant patches in the urban core by *Eriocrania* females emerging from distant sources is improbable, even during peak population years. Therefore, we suggest that colonisation involves multigenerational spread via stepping stones, as described by [67], and that individual patches may act as either sinks or sources in different years.

Unsurprisingly, the occupancy and population density of *Eriocrania* varied among both habitat patches and grid cells, supporting Hypothesis 3. Although our data confirm the island biogeography theory prediction [68] that a larger patch size—as measured by birch abundance—increases the likelihood of *Eriocrania* occupancy, the intercorrelations among patch characteristics hinder the identification of a primary driver of this occupancy based on observational data alone.

Finally, colonisation and extinction appeared well balanced across *Eriocrania*-suitable habitat patches at both spatial and temporal scales. Furthermore, the absence of a detectable directional trend over 40 years disproves Hypothesis 4. This observation is consistent with previous findings from a subarctic region in which *Eriocrania* densities showed no directional trend between 1991 and 2016 [69].

### 4.5. Limitations and Future Research Directions

Despite the exceptional temporal and spatial coverage of this study, several methodological limitations should be noted. Species-level identification of *Eriocrania* was not possible using mine morphology alone, limiting the precision of herbivore data. The large spatial extent precluded complete annual censusing, and changes in social access—such as locked gates and fences—hampered visits to some sites from the early 2000s, restricting close examination of certain birches in some years.

An additional methodological limitation is inherent to the survey approach: the time-restricted search for *Eriocrania* leaf mines in birch foliage may have led to occasional false absences, particularly in large or inaccessible trees. This limitation should be considered when interpreting patch occupancy and extinction risk, and it highlights the need for complementary approaches in future studies.

Future research could build on these findings by incorporating higher-resolution temporal sampling and molecular identification to clarify species-specific responses to urbanisation. Investigating additional environmental drivers—such as microclimate, pollution and urban management—would improve mechanistic understanding of patch dynamics. Comparative studies across multiple plant–herbivore systems and cities would help assess the generality of observed patterns.

The unique insights revealed by this 40-year dataset—insights that could not have been obtained from shorter time series—underscore the value of extended monitoring for understanding ecological processes in heterogeneous urban environments. Such efforts are essential for detecting subtle trends, informing conservation and guiding urban planning.

## 5. Conclusions

Initiated in 1986, this study produced the longest well-replicated dataset to date documenting both spatial and temporal dynamics of a plant–herbivore interaction. It also provided key environmental variables indicative of habitat quality for herbivores across an urbanisation gradient in the large city of St. Petersburg. These data offer rare insights into how organisms respond to the fine-grained environmental heterogeneity characteristic of urban landscapes. We found that soil quality, habitat type and birch group size moderately predicted the risk of host plant and specialist herbivore extinction within a patch, but these factors rarely determined the extinction risk conclusively. In contrast, woody plant cover did not predict either the probability of birch extinction from a patch or the *Eriocrania* population density within a given habitat type. Contrary to earlier conclusions [25], long-term observations indicate that even the urban core does not constitute a true lacuna in the distribution of *Eriocrania* moths. Notably, *Eriocrania* populations have exhibited no directional trend over four decades—an outcome that stands in contrast to the well-documented declines observed for many other insect groups [70,71]. The mechanisms underlying this long-term resilience in stressful urban environments in the face of global change merit further investigation.

## Data Availability

The data are available from Supplementary Materials.

## Supplementary Materials

The following supporting information can be downloaded at: https://www.mdpi.com/article/10.3390/insects17010005/s1, Figure S1: Mine of *Eriocrania* sp. on a downy birch leaf; Figure S2: Habitat types considered in the study; Data S1: Characteristics of habitat patches; Data S2: Characteristics of grid cells.
