# Supplementary material for: Spatial and Temporal Dynamics of Birch-Mining Eriocrania Moths in an Urban Landscape over Four Decades
Source: Insects. 2025 Dec 19;17(1):5. doi: 10.3390/insects17010005 (PMC12842066; doi:10.3390/insects17010005)
Supplement: Supplementary file 1 [file insects-17-00005-s001.zip › insects-4024302-supplementary.pdf]

**Spatial and temporal dynamics of birch-mining *Eriocrania* moths in an urban landscape over four decades**

Mikhail V. Kozlov [a*], Alexandr A. Egorov [b], Elena Valdés-Correcher [c], Vitali Zverev [a]

[a] Department of Biology, University of Turku, 20014 Turku, Finland

[b] Laboratory of Wetland Studies, Institute of Forest Science, Russian Academy of Sciences, Uspenskoe, 143030 Moscow, Russia

[c] Center for Research on Desertification (CIDE), 46113 Moncada, Spain

* Correspondence: mikoz@utu.fi

## Supplementary information

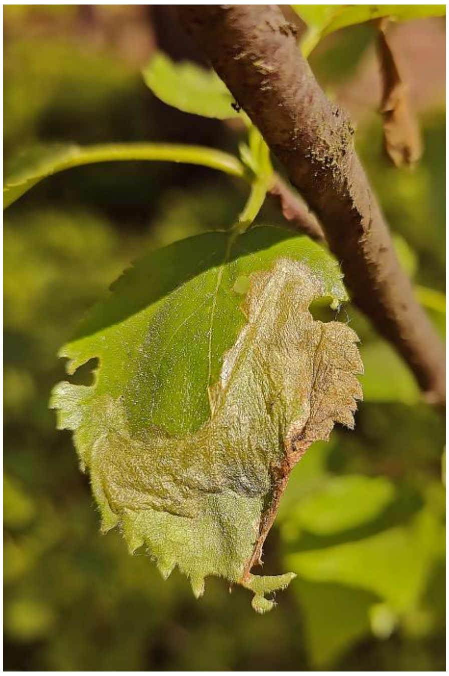

Figure S1. Blotch mine of *Eriocrania* sp. on a downy birch leaf. Photo: V. Zverev.

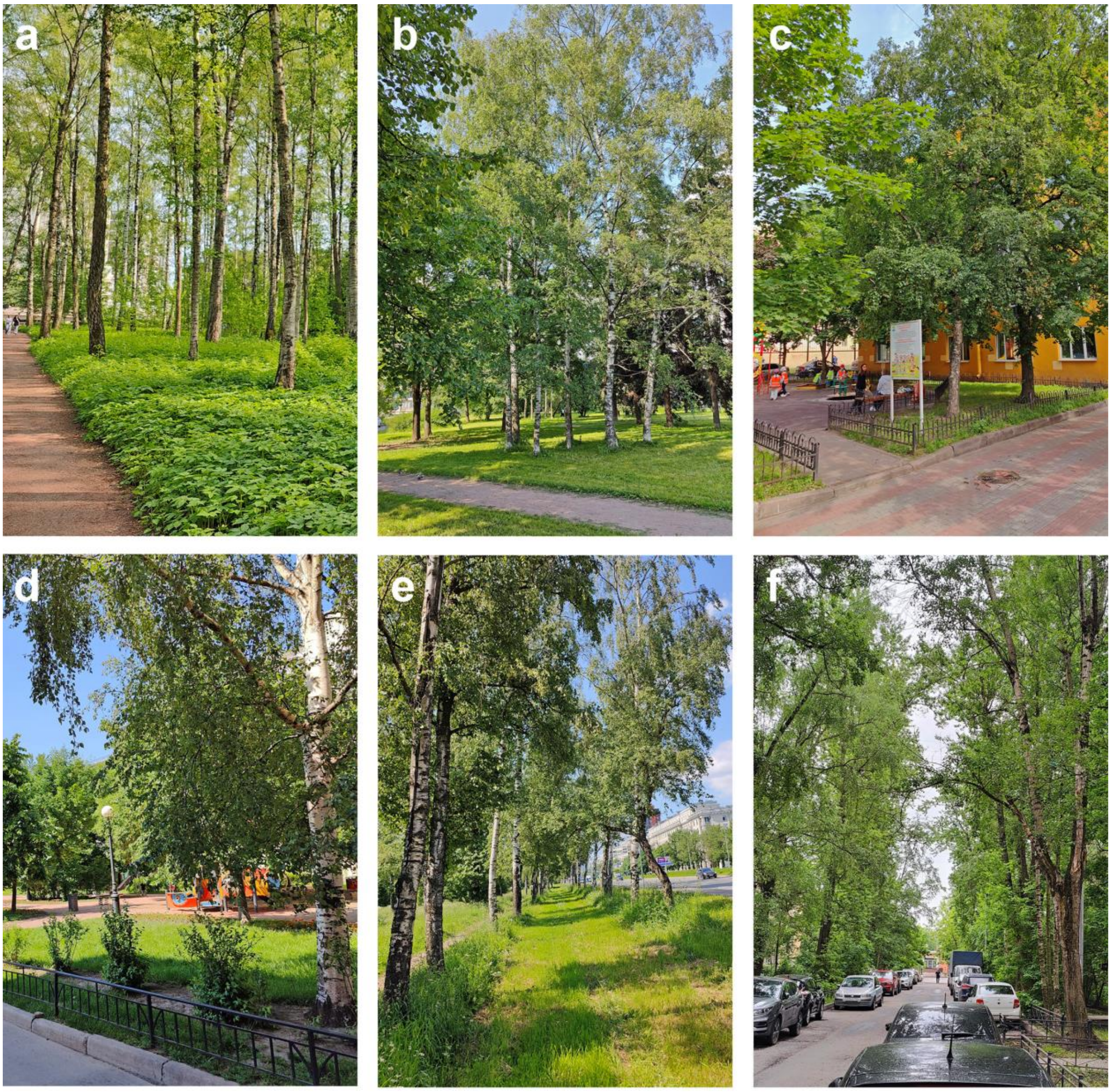


Figure S2. Habitat types considered in the study (a, b – public parks; c, d – public gardens; e, f – roadsides). Photo: A. Egorov.

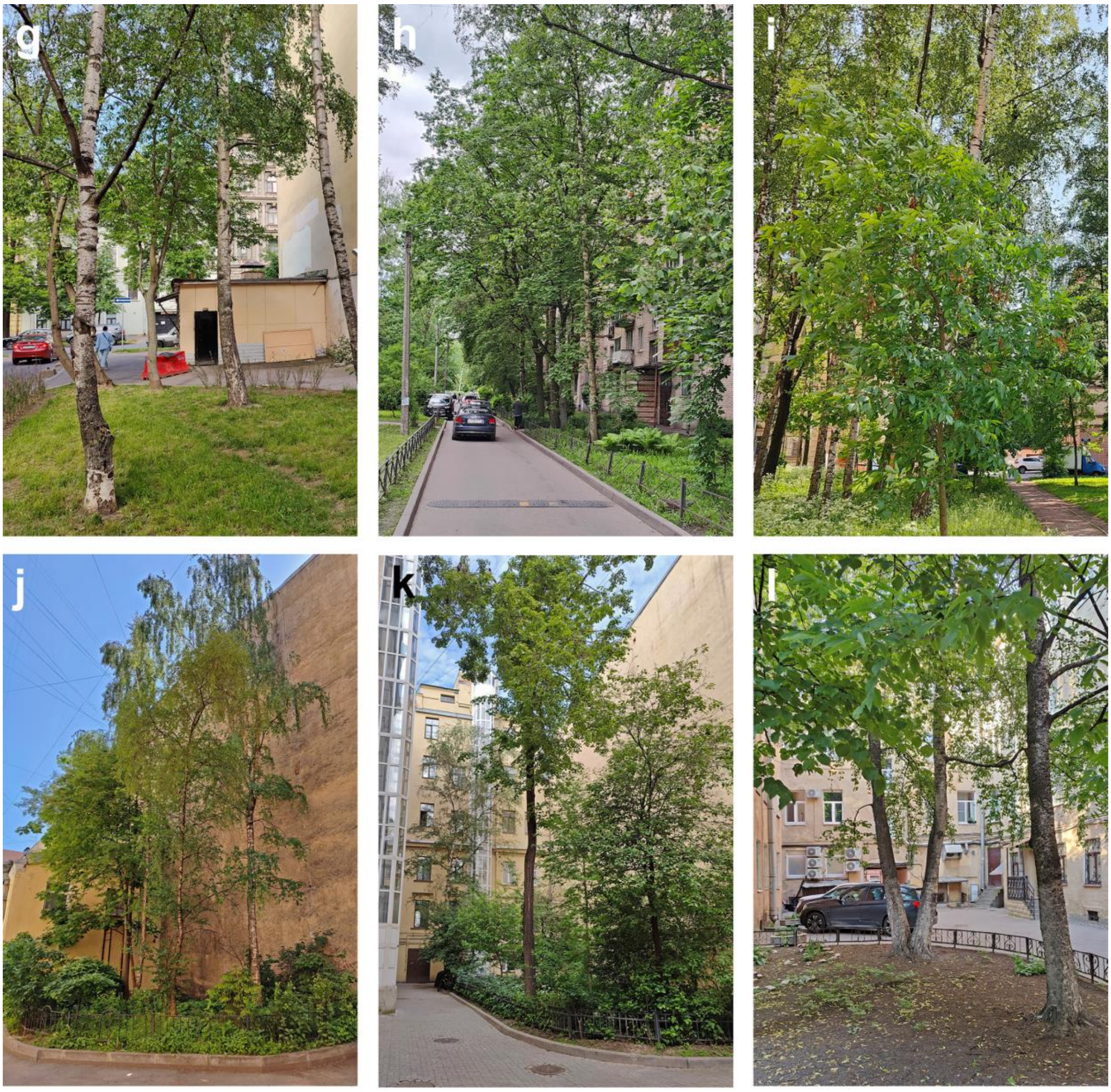


Figure S2 (continued). Habitat types considered in the study (g, h, i – open courtyards; j, k, l – enclosed courtyards). Photo: A. Egorov.

**Data S1**. Characteristics of habitat patches.

Column 1: Identifier (1 to 150).
Column 2: Latitude (N) of patch centre.
Column 3: Longitude (E) of patch centre.
Column 4: Distance from the city centre (the Palace Square), km.
Column 5: Habitat type in 1986.
Column 6: Habitat type in 2000.
Column 7: Habitat type in 2012.
Column 8: Habitat type in 2025.
Column 9: Presence of soil type (A, asphalt; G, gravel; L, loosened; S, sand; T, trampled; U, undisturbed) in 1986.
Column 10: Presence of soil type (A, asphalt; G, gravel; L, loosened; S, sand; T, trampled; U, undisturbed) in 2000.
Column 11: Presence of soil type (A, asphalt; G, gravel; L, loosened; S, sand; T, trampled; U, undisturbed) in 2012.
Column 12: Presence of soil type (A, asphalt; G, gravel; L, loosened; S, sand; T, trampled; U, undisturbed) in 2025.
Column 13: Woody plant cover within a 30-m radius around habitat patch centre in 1984, %.
Column 14: Woody plant cover within a 30-m radius around habitat patch centre in 2023, % (measured only in patches, for which data of 1984 are available).
Column 15: Birch group size in 1986, number of trees or size category (S, small, 1–3 trees; M, medium, 4–10 trees; L, large, >10 trees).
Column 16: Birch group size in 2000, number of trees or size category (S, small, 1–3 trees; M, medium, 4–10 trees; L, large, >10 trees).
Column 17: Birch group size in 2012, number of trees or size category (S, small, 1–3 trees; M, medium, 4–10 trees; L, large, >10 trees).
Column 18: Birch group size in 2025, number of trees or size category (S, small, 1–3 trees; M, medium, 4–10 trees; L, large, >10 trees).
Column 19: Path and birch fate in 2025 relative to 1984 (absent, birches were absent throughout the entire observation period, despite habitats remain suitable for birches; destroyed, habitats were irreversibly destroyed during the observation period, causing loss of vegetation; lost, birches occurred in 1986 but went extinct later, despite habitats remain suitable for birches; missing, patches inaccessible in 2025; planted, patches where birches were absent in 1986 but planted later; present, patches where birches persisted from 1986 to 2025, including those were birches were planted following extinction).
Column 20: Number of *Betula pendula* trees/number of *B. pubescens* trees (data of 2025).
Column 21: Median height of birches in 1986, m.
Column 22: Median height of birches in 2000, m.
Column 23: Median height of birches in 2012, m.
Column 24: Median height of birches in 2025, m.
Column 25: *Eriocrania* density in 1986 (0, no mines; 1, low density; 2, high density; na, mine density could not be assessed; nv, patch had not been visited).
Column 26: *Eriocrania* density in 1990 (0, no mines; 1, low density; 2, high density; na, mine density could not be assessed; nv, patch had not been visited).
Column 27: *Eriocrania* density in 2000 (0, no mines; 1, low density; 2, high density; na, mine density could not be assessed; nv, patch had not been visited).
Column 28: *Eriocrania* density in 2001 (0, no mines; 1, low density; 2, high density; na, mine density could not be assessed; nv, patch had not been visited).

Column 29: *Eriocrania* density in 2002 (0, no mines; 1, low density; 2, high density; na, mine density could not be assessed; nv, patch had not been visited).
Column 30: *Eriocrania* density in 2003 (0, no mines; 1, low density; 2, high density; na, mine density could not be assessed; nv, patch had not been visited).
Column 31: *Eriocrania* density in 2004 (0, no mines; 1, low density; 2, high density; na, mine density could not be assessed; nv, patch had not been visited).
Column 32: *Eriocrania* density in 2005 (0, no mines; 1, low density; 2, high density; na, mine density could not be assessed; nv, patch had not been visited).
Column 33: *Eriocrania* density in 2006 (0, no mines; 1, low density; 2, high density; na, mine density could not be assessed; nv, patch had not been visited).
Column 34: *Eriocrania* density in 2007 (0, no mines; 1, low density; 2, high density; na, mine density could not be assessed; nv, patch had not been visited).
Column 35: *Eriocrania* density in 2008 (0, no mines; 1, low density; 2, high density; na, mine density could not be assessed; nv, patch had not been visited).
Column 36: *Eriocrania* density in 2009 (0, no mines; 1, low density; 2, high density; na, mine density could not be assessed; nv, patch had not been visited).
Column 37: *Eriocrania* density in 2010 (0, no mines; 1, low density; 2, high density; na, mine density could not be assessed; nv, patch had not been visited).
Column 38: *Eriocrania* density in 2011 (0, no mines; 1, low density; 2, high density; na, mine density could not be assessed; nv, patch had not been visited).
Column 39: *Eriocrania* density in 2012 (0, no mines; 1, low density; 2, high density; na, mine density could not be assessed; nv, patch had not been visited).
Column 40: *Eriocrania* density in 2023 (0, no mines; 1, low density; 2, high density; na, mine density could not be assessed; nv, patch had not been visited).
Column 41: *Eriocrania* density in 2024 (0, no mines; 1, low density; 2, high density; na, mine density could not be assessed; nv, patch had not been visited).
Column 42: *Eriocrania* density in 2025 (0, no mines; 1, low density; 2, high density; na, mine density could not be assessed; nv, patch had not been visited).

```
1,59.921111,30.335000,2.2817,park,park,park,park,TU,TU,TU,TU,14,30,48,70,L,L,L,5+,Present,5/0,12,0,12,0,14,0,14,0,1,nv,1,2,2,2,2,1,1,1,1,2,1,1,1,1,nv,0,,,
2,59.925536,30.341944,2.1134,enclosed_courtyard,enclosed_courtyard,enclosed_courtyard,enclosed_courtyard,U,AT,T,T,0,35,0,00,1,1,1,0,Lost,.,5,0,7,0,7,0,.,0,0,1,0,nv,nv,nv,nv,nv,nv,nv,nv,nv,nv,nv,nv,nv,nv,,,
3,59.926329,30.344444,2.1542,enclosed_courtyard,enclosed_courtyard,enclosed_courtyard,enclosed_courtyard,ALU,ALU,ALU,LTU,3,17,1,13,4,0,0,0,Lost,.,10,0,.,.,.,16,5,2,nv,nv,nv,nv,nv,nv,nv,nv,nv,nv,nv,nv,nv,nv,nv,nv,nv,,,
4,59.927154,30.345893,2.1579,enclosed_courtyard,enclosed_courtyard,enclosed_courtyard,enclosed_courtyard,L,AT,ALTU,ALU,6,19,15,29,1,2,2,2,Present,0/2,4,0,7,0,16,0,17,5,0,0,1,1,2,1,1,0,0,na,nv,nv,nv,nv,na,nv,nv,0,,,
5,59.931115,30.349404,2.0930,enclosed_courtyard,enclosed_courtyard,enclosed_courtyard,enclosed_courtyard,L,AT,LU,ATU,0,56,0,00,2,3,3,3,Present,3/0,3,0,5,0,16,5,16,5,0,nv,0,1,2,2,2,0,1,na,nv,nv,nv,nv,na,nv,nv,0,,,
6,59.935739,30.349714,1.9466,open_courtyard,open_courtyard,open_courtyard,open_courtyard,AT,ATU,AT,AU,4,66,4,33,5,5,10,1+,Present,1/0,10,0,16,5,10,5,17,0,1,nv,na,1,2,2,2,0,na,na,nv,nv,nv,nv,0,nv,nv,na,,,
7,59.93977,30.34915,1.8783,enclosed_courtyard,enclosed_courtyard,enclosed_courtyard,enclosed_courtyard,LU,ALU,LU,ATU,4,88,22,69,2,3,.,1,Present,1/0,2,0,3,0,3,0,6,0,2,0,1,1,2,2,2,0,0,0,0,0,0,na,na,nv,nv,1,,,
8,59.941944,30.348889,1.8879,enclosed_courtyard,enclosed_courtyard,built_area,built_area,S,AST,.,S,27,01,0,00,S,S,.,.,Destroyed,.,15,0,15,0,.,.,0,0,1,1,2,1,2,na,nv,nv,nv,nv,nv,nv,nv,nv,nv,nv,,,
9,59.947060,30.34429,1.8282,enclosed_courtyard,enclosed_courtyard,enclosed_courtyard,enclosed_courtyard,L,L,L,AT,7,28,14,96,1,1,1,1,Present,1/0,3,0,3,0,.,15,0,0,nv,na,nv,nv,nv,nv,nv,nv,nv,nv,nv,nv,nv,nv,nv,nv,0,,,
10,59.946204,30.340249,1.5830,enclosed_courtyard,enclosed_courtyard,enclosed_courtyard,enclosed_courtyard,U,U,U,U,17,73,13,06,M,S,S,1,Present,0/1,15,0,17,5,15,0,13,5,1,nv,1,2,2,2,2,0,1,na,nv,nv,nv,nv,0,nv,nv,0,,,
11,59.944769,30.339481,1.4711,roadside,roadside,roadside,roadside,U,U,U,ASTU,9,03,2,64,S,0,0,0,Lost,.,2,0,.,.,.,.,0,nv,nv,nv,nv,nv,nv,nv,nv,nv,nv,nv,nv,nv,nv,nv,nv,nv,,,
12,59.944692,30.335570,1.2707,public_garden,public_garden,public_garden,public_garden,TU,TU,TU,TU,98,77,69,66,0,0,0,0,Absent,.,.,.,.,.,.,nv,nv,nv,nv,nv,nv,nv,nv,nv,nv,nv,nv,nv,nv,nv,nv,nv,nv,,,
13,59.95444,30.35556,2.8107,public_garden,public_garden,public_garden,public_garden,TU,TU,TU,TU,5,73,4,24,0,0,0,0,Absent,.,.,.,.,.,.,nv,nv,nv,nv,nv,nv,nv,nv,nv,nv,nv,nv,nv,nv,nv,nv,nv,nv,,,
14,59.93919,30.33933,1.3238,public_garden,public_garden,public_garden,public_garden,U,U,U,TU,63,05,40,42,0,4,4,4,Planted,4/0,.,11,0,12,5,12,5,nv,nv,1,nv,nv,nv,nv,nv,nv,nv,nv,nv,nv,nv,nv,nv,0,,,
15,59.93722,30.33167,0.9182,public_garden,public_garden,public_garden,public_garden,GU,GU,GU,AGLU,37,02,47,06,0,0,0,0,Absent,.,.,.,.,.,.,nv,nv,nv,nv,nv,nv,nv,nv,nv,nv,nv,nv,nv,nv,nv,nv,nv,nv,,,
16,59.933035,30.337136,1.3807,public_garden,public_garden,public_garden,public_garden,GU,GU,GU,AGLU,46,92,41,03,0,0,0,0,Absent,.,.,.,.,.,.,nv,nv,nv,nv,nv,nv,nv,nv,nv,nv,nv,nv,nv,nv,nv,nv,nv,nv,,,
17,59.932824,30.340708,1.5693,public_garden,public_garden,public_garden,public_garden,ALU,ALU,ALU,AU,22,66,24,13,M,M,3,2,Present,2/0,15,0,15,0,18,0,20,0,1,nv,1,2,2,2,2,0,0,0,2,1,0,1,na,nv,nv,0,,,
18,59.931878,30.358074,2.5144,enclosed_courtyard,enclosed_courtyard,enclosed_courtyard,enclosed_courtyard,AT,AU,AT,AT,0,77,6,07,2,2,2,2,Present,2/0,12,0,12,0,18,0,24,0,0,nv,0,1,2,2,2,na,0,0,na,na,na,na,0,nv,nv,0,,,
```

```
19,59.934181,30.354316,2.2320,public_garden,public_garden,public_garden,public_garden,U,ATU,TU,AU,30,83,37,27,7,6,6,6,Present,0/3,10,0,10,0,16,5,15,5,1,nv,1,1,2,2,2,0,0,2,0,1,1,1,0,0,nv,0,,,
20,59.936253,30.354313,2.1921,enclosed_courtyard,enclosed_courtyard,enclosed_courtyard,enclosed_courtyard,L,AT,L,AU,3,60,10,87,S,5,M,3,Present,0/3,10,0,10,0,10,0,17,0,0,nv,1,1,2,2,2,0,1,0,na,na,na,na,na,nv,nv,0,,,
21,59.940897,30.35456,2.1904,enclosed_courtyard,enclosed_courtyard,enclosed_courtyard,enclosed_courtyard,TU,TU,TU,.,21,23,25,19,1,1,1,.,Missing,.,15,0,15,0,15,0,.,0,nv,na,na,2,2,1,na,na,nv,na,na,na,na,na,nv,nv,nv,,,
22,59.94139,30.35458,2.1970,enclosed_courtyard,enclosed_courtyard,enclosed_courtyard,enclosed_courtyard,ST,ST,AT,T,5,18,10,02,2,2,2,2,Present,3/0,12,0,12,0,16,0,15,0,0,nv,1,1,2,2,1,0,0,0,1,1,0,1,0,nv,nv,0,,,
23,60.03925,30.40259,12.1663,roadside,roadside,roadside,roadside,AU,AU,ATU,AU,1,70,0,00,S,3,4,0,Lost,.,6,0,15,0,14,0,14,0,1,nv,1,nv,nv,nv,nv,nv,nv,nv,nv,nv,nv,nv,0,0,0,nv,,,
24,60.04025,30.39835,12.1751,roadside,roadside,roadside,roadside,AT,ATU,AT,AT,4,00,14,15,M,M,15+,18,Present,16/2,6,0,8,5,12,5,12,5,1,nv,1,nv,nv,nv,nv,nv,nv,nv,nv,nv,nv,nv,1,0,0,0,,,
25,60.00371,30.31920,7.1874,park,park,park,park,TU,TU,.,TU,99,35,93,84,L,L,.,25+,Present,0/25,.,.,.,.,18,0,2,nv,nv,nv,nv,nv,nv,nv,nv,nv,nv,nv,nv,nv,nv,nv,nv,2,,,
26,60.02943,30.34622,10.1929,park,park,park,park,TU,TU,.,TU,84,15,92,86,L,L,.,25+,Present,0/25,.,.,.,.,20,0,2,nv,nv,nv,nv,nv,nv,nv,nv,nv,nv,nv,nv,nv,nv,nv,nv,2,,,
27,60.01036,30.34878,8.1397,public_garden,public_garden,public_garden,public_garden,T,TU,GTU,GTU,34,13,31,02,M,M,8,8,Present,8/0,15,0,15,0,16,5,16,5,1,nv,1,nv,nv,nv,nv,nv,nv,nv,nv,nv,nv,nv,1,0,0,0,,,
28,60.01022,30.34691,8.1011,roadside,roadside,roadside,roadside,U,TU,ATU,ATU,8,09,34,72,M,M,M,9,Present,9/0,10,0,16,0,16,5,16,5,2,nv,1,nv,nv,nv,nv,nv,nv,nv,nv,nv,nv,nv,0,1,0,0,,,
29,60.00975,30.34598,8.0388,roadside,roadside,roadside,roadside,U,U,TU,TU,42,66,26,83,L,L,L,11,Present,10/0,15,0,16,5,18,0,19,0,2,nv,1,nv,nv,nv,nv,nv,nv,nv,nv,nv,nv,nv,0,1,0,0,,,
30,60.00825,30.34128,7.8228,roadside,roadside,roadside,roadside,U,U,U,U,38,61,22,05,M,M,M,10+,Present,11/0,15,0,17,5,17,5,17,5,1,nv,2,nv,nv,nv,nv,nv,nv,nv,nv,nv,nv,nv,1,0,0,0,,,
31,60.00694,30.33722,7.6401,roadside,roadside,roadside,roadside,AU,AU,AU,ATU,13,23,0,70,L,L,0,0,Lost,.,10,0,15,0,.,.,.,1,nv,2,nv,nv,nv,nv,nv,nv,nv,nv,nv,nv,nv,nv,nv,nv,,,
32,60.00235,30.32712,7.0622,public_garden,public_garden,public_garden,public_garden,TU,TU,TU,TU,25,68,47,60,M,M,6,4,Present,4/0,5,0,5,0,11,5,14,0,1,nv,2,nv,nv,nv,nv,nv,nv,nv,nv,nv,nv,nv,1,1,0,0,,,
33,59.99720,30.32341,6.4743,roadside,roadside,roadside,roadside,U,U,AT,AT,22,81,33,41,M,M,3,3,Present,3/0,15,0,20,0,20,0,20,0,0,nv,1,nv,nv,nv,nv,nv,nv,nv,nv,nv,nv,1,nv,0,0,,,
34,59.99689,30.32256,6.4369,roadside,roadside,roadside,roadside,U,U,TU,AT,18,15,39,69,S,M,3,3,Present,3/0,3,0,12,0,16,5,20,0,1,nv,1,nv,nv,nv,nv,nv,nv,nv,nv,nv,nv,nv,0,nv,0,0,,,
35,59.97417,30.30194,3.9744,open_courtyard,open_courtyard,open_courtyard,open_courtyard,U,U,U,U,69,85,35,57,1,0,0,0,Lost,.,10,0,.,.,.,.,0,nv,nv,nv,nv,nv,nv,nv,nv,nv,nv,nv,nv,nv,nv,nv,nv,nv,,,
36,59.96537,30.30869,2.9445,public_garden,public_garden,public_garden,public_garden,TU,AT,LTU,AT,22,09,27,07,M,1,1,1,Present,0/1,8,0,12,0,10,0,19,0,0,nv,2,1,2,2,1,0,0,0,1,2,1,1,1,0,nv,0,,,
37,59.95923,30.30015,2.4018,roadside,roadside,roadside,roadside,ST,TU,ST,AST,32,12,12,01,S,3,2,2,Present,0/1,15,0,15,0,15,0,15,0,0,nv,1,1,2,2,2,0,0,0,1,1,1,1,0,nv,nv,0,,,
38,59.96004,30.2992,2.5054,roadside,roadside,roadside,roadside,AT,AT,ATU,ATU,32,49,19,13,1,1,1,1,Present,1/1,15,0,15,0,15,0,15,0,0,nv,1,1,2,2,1,0,0,0,1,1,1,0,0,nv,nv,0,,,
39,59.95194,30.29056,2.0123,public_garden,public_garden,public_garden,public_garden,TU,TU,TU,ATU,25,47,6,58,1,1,0,0,Lost,.,5,0,15,0,.,.,.,1,nv,1,2,2,1,0,0,1,0,nv,nv,nv,nv,nv,nv,nv,nv,,,
40,59.951809,30.293003,1.9066,public_garden,public_garden,public_garden,public_garden,TU,TU,TU,TU,66,34,50,77,L,L,L,L,Present,2/0,15,0,15,0,18,0,18,5,1,nv,nv,nv,nv,nv,nv,nv,nv,nv,nv,nv,nv,0,nv,nv,0,,,
41,59.85801,30.32017,9.0377,public_garden,public_garden,public_garden,public_garden,U,U,U,U,.,.,.,L,L,30+,30+,Present,21/0,17,5,17,5,17,5,17,5,1,nv,2,nv,nv,nv,nv,nv,nv,nv,nv,nv,nv,nv,0,nv,1,0,,,
42,59.85889,30.32859,8.9653,open_courtyard,open_courtyard,open_courtyard,open_courtyard,T,TU,ATU,ATU,.,.,.,S,2,2,2,Present,1/2,8,0,8,0,16,5,18,5,0,nv,2,nv,nv,nv,nv,nv,nv,nv,nv,nv,nv,nv,1,nv,1,1,,,
43,59.86235,30.32857,8.5813,public_garden,public_garden,public_garden,public_garden,U,U,TU,TU,.,.,.,8,8,8,8,Present,8/0,8,0,8,0,15,0,15,0,1,nv,2,nv,nv,nv,nv,nv,nv,nv,nv,nv,nv,nv,1,nv,2,1,,,
44,59.87116,30.33114,7.6195,park,park,park,park,U,U,LTU,TU,.,.,.,M,M,M,6,Present,6/0,11,5,11,5,16,5,16,5,1,nv,2,nv,nv,nv,nv,nv,nv,nv,nv,nv,nv,1,nv,0,1,,,
45,59.87065,30.3064,7.6458,open_courtyard,open_courtyard,open_courtyard,open_courtyard,U,U,U,U,.,.,.,1,2,2,0,Lost,.,15,0,15,0,15,0,.,1,nv,2,nv,nv,nv,nv,nv,nv,nv,nv,nv,nv,nv,nv,nv,nv,nv,,,
46,59.87102,30.30506,7.6104,park,park,park,park,U,U,TU,TU,.,.,.,2,1,1,1,Present,1/0,15,0,15,0,18,0,18,0,1,nv,2,nv,nv,nv,nv,nv,nv,nv,nv,nv,nv,0,nv,0,0,,,
47,59.86911,30.30507,7.8223,park,park,park,park,LU,AU,AU,TU,.,.,.,1,3,2,2,Present,5/0,15,0,15,0,18,0,16,5,0,nv,2,nv,nv,nv,nv,nv,nv,nv,nv,nv,nv,2,nv,0,0,,,
48,59.87215,30.30732,7.4757,open_courtyard,open_courtyard,open_courtyard,open_courtyard,TU,TU,ATU,ATU,.,.,.,15+,15+,15+,15+,Present,7/3,15,0,15,0,16,5,15,0,1,nv,1,nv,nv,nv,nv,nv,nv,nv,nv,nv,nv,1,nv,1,1,,,
49,59.87363,30.30763,7.3102,open_courtyard,open_courtyard,open_courtyard,open_courtyard,U,U,U,TU,.,.,.,M,0,0,0,Lost,.,15,0,.,.,.,.,1,nv,nv,nv,nv,nv,nv,nv,nv,nv,nv,nv,nv,nv,nv,nv,nv,nv,,,
50,59.87507,30.32645,7.1603,open_courtyard,open_courtyard,open_courtyard,open_courtyard,U,U,TU,TU,.,.,.,L,L,55,40,Present,40/0,16,5,16,5,19,0,19,0,0,nv,1,nv,nv,nv,nv,nv,nv,nv,nv,nv,nv,nv,0,nv,0,0,,,
51,59.87634,30.33483,7.0753,roadside,roadside,roadside,roadside,ST,ST,ASTU,ATU,.,.,.,L,L,33,29,Present,29/0,15,0,15,0,17,5,17,5,0,nv,1,nv,nv,nv,nv,nv,nv,nv,nv,nv,nv,1,nv,0,0,,,
52,59.88099,30.36120,6.9624,park,park,park,park,U,U,.,TU,.,.,.,L,L,.,5+,Present,2/3,15,0,15,0,.,17,5,2,nv,nv,nv,nv,nv,nv,nv,nv,nv,nv,nv,nv,nv,nv,nv,nv,0,,,
53,59.89850,30.32435,4.5524,park,park,park,park,U,U,.,U,.,.,.,L,L,.,L,Present,1/0,17,5,17,5,.,17,5,2,nv,nv,nv,nv,nv,nv,nv,nv,nv,nv,nv,nv,nv,nv,nv,nv,1,,,
54,59.913033,30.319396,2.9162,public_garden,public_garden,public_garden,public_garden,TU,TU,TU,TU,71,27,50,37,0,0,0,0,Absent,.,.,.,.,.,.,nv,nv,nv,nv,nv,nv,nv,nv,nv,nv,nv,nv,nv,nv,nv,nv,nv,nv,,,
55,59.916512,30.313113,2.5599,roadside,roadside,roadside,roadside,T,AT,T,AU,10,37,23,62,2,1,1,1,Present,0/1,2,0,6,0,6,0,6,0,2,2,1,2,2,2,0,0,0,0,1,1,1,1,0,nv,nv,0,,,
56,59.91824,30.31221,2.3385,park,park,park,park,TU,TU,TU,TU,50,32,18,50,0,6,6,6,Planted,6/0,.,.,.,.,20,5,nv,nv,nv,nv,nv,nv,nv,nv,nv,nv,nv,nv,nv,nv,nv,nv,0,,,
57,59.916667,30.306944,2.5543,public_garden,public_garden,public_garden,public_garden,TU,TU,TU,ATU,34,38,19,16,2,0,0,0,Lost,.,20,0,.,.,.,.,na,nv,nv,nv,nv,nv,nv,nv,nv,nv,nv,nv,nv,nv,nv,nv,nv,nv,,,
58,59.924671,30.313114,1.6209,roadside,roadside,roadside,roadside,U,U,U,U,23,57,15,23,1,0,0,0,Lost,.,16,0,.,.,.,.,1,nv,nv,1,nv,nv,nv,nv,nv,nv,nv,nv,nv,nv,nv,nv,nv,nv,,,
59,59.923969,30.311654,1.7081,public_garden,public_garden,public_garden,public_garden,U,TU,U,U,43,85,33,67,M,M,M,M,Lost,.,20,0,20,0,20,0,.,na,nv,2,1,2,2,2,0,0,0,1,1,0,0,1,nv,nv,0,,,
60,59.84135,30.24772,11.5454,open_courtyard,open_courtyard,open_courtyard,open_courtyard,U,U,LTU,LTU,.,.,.,L,L,15+,15,Present,3/7,11,5,11,5,17,5,17,5,1,nv,2,nv,nv,nv,nv,nv,nv,nv,nv,nv,nv,nv,0,nv,0,1,,,
61,59.841944,30.246111,11.5137,public_garden,public_garden,public_garden,public_garden,U,U,.,U,.,.,.,L,L,.,10+,Present,10/0,11,5,11,5,.,11,5,1,nv,nv,nv,nv,nv,nv,nv,nv,nv,nv,nv,nv,nv,nv,nv,1,,,
```

62,59.83154,30.23491,12.8192,park,park,park,park,U,U,.,TU,.,.,L,L,.,10,Present,2/8,12,5,12,5,.,12,5,1,nv,nv,nv,nv,nv,nv,nv,nv,nv,nv,nv,nv,nv,nv,nv,1,,,
63,59.83415,30.13674,15.4427,park,park,park,park,U,U,.,TU,.,.,L,L,.,25+,Present,0/10,15,0,15,0,.,15,0,1,nv,nv,nv,nv,nv,nv,nv,nv,nv,nv,nv,nv,nv,nv,nv,2,,,
64,59.83639,30.12808,15.5818,open_courtyard,open_courtyard,open_courtyard,open_courtyard,TU,TU,TU,AU,.,.,L,1,2,2,Present,1/0,8,0,12,0,13,5,13,5,0,nv,2,nv,nv,nv,nv,nv,nv,nv,nv,nv,nv,1,nv,0,1,,,
65,59.84153,30.13156,15.0306,open_courtyard,open_courtyard,open_courtyard,open_courtyard,U,U,UA,AU,.,.,M,M,4+,4+,Present,4/0,7,0,7,0,17,0,17,0,0,nv,2,nv,nv,nv,nv,nv,nv,nv,nv,nv,nv,nv,0,nv,0,0,,,
66,59.84446,30.14482,14.2822,open_courtyard,open_courtyard,open_courtyard,open_courtyard,U,U,U,ATU,.,.,M,M,3,3,Present,3/0,4,0,4,0,16,5,16,5,1,nv,na,nv,nv,nv,nv,nv,nv,nv,nv,nv,nv,nv,0,nv,0,0,,,
67,59.84545,30.13699,14.5040,park,park,park,park,U,U,U,TU,.,.,L,L,L,8,Present,7/1,12,5,12,5,12,5,15,0,0,nv,2,nv,nv,nv,nv,nv,nv,nv,nv,nv,nv,1,nv,2,1,,,
68,59.84200,30.15712,14.0341,park,park,park,park,TU,.,.,TU,.,.,M,.,.,25+,Present,4/6,12,5,.,.,15,0,1,nv,nv,nv,nv,nv,nv,nv,nv,nv,nv,nv,nv,nv,nv,nv,1,,,
69,59.84883,30.17364,12.8572,park,park,park,park,U,.,U,U,.,.,M,.,5+,23,Present,23/0,12,5,.,16,5,12,0,na,nv,nv,nv,nv,nv,nv,nv,nv,nv,nv,nv,nv,1,nv,0,0,,,
70,59.85509,30.2494,10.0801,open_courtyard,open_courtyard,open_courtyard,open_courtyard,U,U,LTU,LTU,.,.,S,S,0,22,Present,4/18,12,0,12,0,12,0,12,0,1,nv,1,nv,nv,nv,nv,nv,nv,nv,nv,nv,nv,nv,nv,nv,nv,0,,,
71,59.85658,30.25035,9.9062,open_courtyard,open_courtyard,open_courtyard,open_courtyard,U,U,ATU,ATU,.,.,M,M,2,2,Present,2/0,3,0,3,0,18,0,18,0,1,nv,2,nv,nv,nv,nv,nv,nv,nv,nv,nv,nv,nv,0,nv,0,0,,,
72,59.86382,30.26242,8.9111,park,park,park,park,U,STU,STU,STU,.,.,M,M,M,+,Present,5/0,12,5,18,0,19,0,19,0,na,nv,1,nv,nv,nv,nv,nv,nv,nv,nv,nv,nv,nv,na,nv,0,0,,,
73,59.86911,30.26095,8.3894,open_courtyard,open_courtyard,open_courtyard,open_courtyard,ST,ST,ST,ST,.,.,L,0,0,0,Lost,.,18,0,.,.,.,0,nv,nv,nv,nv,nv,nv,nv,nv,nv,nv,nv,nv,nv,nv,nv,nv,,,
74,59.87074,30.26126,8.2144,open_courtyard,open_courtyard,open_courtyard,open_courtyard,ST,ST,ST,ST,.,.,1,1,1,1,Present,1/0,18,0,18,0,18,0,18,0,0,nv,0,nv,nv,nv,nv,nv,nv,nv,nv,nv,nv,nv,nv,nv,1,0,,,
75,59.87318,30.25841,8.0266,roadside,roadside,roadside,roadside,U,.,AL,AL,.,.,4,.,1,1,Present,1/0,3,0,.,5,0,5,0,0,nv,nv,nv,nv,nv,nv,nv,nv,nv,nv,nv,nv,nv,0,nv,0,0,,,
76,59.87287,30.25644,8.1033,roadside,roadside,roadside,roadside,U,U,AU,ATU,.,.,L,L,1,3,Present,3/0,12,5,12,5,15,0,15,0,1,nv,2,nv,nv,nv,nv,nv,nv,nv,nv,nv,nv,nv,1,nv,0,0,,,
77,59.883438,30.265118,6.8301,roadside,roadside,road,road,AT,.,.,ASU,.,.,4,0,.,.,Destroyed,0/1,15,0,.,.,18,0,0,nv,nv,nv,nv,nv,nv,nv,nv,nv,nv,nv,nv,nv,nv,nv,nv,nv,,,
78,59.885225,30.267534,6.5926,public_garden,public_garden,public_garden,public_garden,U,U,U,TU,.,.,1,1,0,0,Lost,.,15,0,15,0,.,15,0,1,nv,0,nv,nv,nv,nv,nv,nv,nv,nv,nv,nv,nv,nv,nv,nv,nv,,,
79,59.886923,30.268654,6.3944,roadside,roadside,roadside,roadside,AT,AT,AT,AT,.,.,2,2,2,2,Present,0/2,15,0,15,0,15,0,15,0,2,nv,1,nv,nv,nv,nv,nv,nv,nv,nv,nv,nv,1,nv,nv,0,,,
80,59.88811,30.27166,6.2047,open_courtyard,open_courtyard,open_courtyard,open_courtyard,U,TU,ATU,ATU,.,.,M,L,8,5,Present,0/5,3,0,3,0,18,0,18,0,0,nv,2,nv,nv,nv,nv,nv,nv,nv,nv,nv,nv,nv,0,nv,nv,1,,,
81,59.89228,30.27511,5.7015,park,park,park,park,TU,TU,TU,ATU,.,.,M,2,2,2,Present,2/2,17,5,20,0,20,0,25,0,1,nv,2,nv,nv,nv,nv,nv,nv,nv,nv,nv,nv,0,nv,nv,0,,,
82,59.89867,30.26975,5.1193,roadside,roadside,roadside,roadside,U,TU,TU,SU,.,.,M,M,3,3,Present,0/3,20,0,20,0,20,0,20,0,1,nv,2,nv,nv,nv,nv,nv,nv,nv,nv,nv,nv,0,nv,nv,0,,,
83,59.90220,30.26735,4.9392,park,park,park,park,TU,TU,.,TU,.,.,L,L,.,25+,Present,9/1,12,5,12,5,.,12,5,1,nv,nv,nv,nv,nv,nv,nv,nv,nv,nv,nv,nv,nv,nv,nv,1,,,
84,59.95842,30.41518,5.9935,roadside,roadside,roadside,roadside,U,U,AU,AU,3,87,10,13,5,5,5,5,Present,3/2,15,0,18,0,17,0,20,0,1,nv,1,nv,nv,nv,nv,nv,nv,nv,nv,nv,nv,0,nv,nv,0,,,
85,59.96084,30.35357,3.2158,public_garden,public_garden,public_garden,public_garden,U,U,AU,ATU,46,46,44,83,6,6,6,6,Present,6/0,15,0,15,0,18,0,18,0,1,nv,1,nv,nv,nv,nv,nv,nv,nv,nv,nv,nv,1,nv,2,0,,,
86,59.97115,30.34783,3.9907,public_garden,public_garden,asphalted_area,asphalted_area,U,U,AU,AU,3,80,0,49,L,L,0,0,Destroyed,.,17,5,17,5,.,.,1,nv,2,nv,nv,nv,nv,nv,nv,nv,nv,nv,nv,nv,nv,nv,nv,nv,,,
87,59.97309,30.34687,4.1615,roadside,roadside,roadside,roadside,ST,ST,AT,AT,56,29,31,07,S,2,2,2,Present,2/0,12,0,6,0,8,0,18,0,0,nv,1,nv,nv,nv,nv,nv,nv,nv,nv,nv,nv,nv,1,nv,0,0,,,
88,59.97796,30.34019,4.5312,roadside,roadside,roadside,roadside,TU,TU,TU,TU,33,44,28,52,L,0,0,0,Lost,.,15,0,.,.,.,1,nv,nv,nv,nv,nv,nv,nv,nv,nv,nv,nv,nv,nv,nv,nv,nv,,,
89,59.97899,30.36093,5.1096,roadside,roadside,roadside,roadside,TU,TU,TU,TU,25,44,36,20,L,L,20+,20+,Present,20/0,3,0,3,0,15,5,20,0,0,nv,1,nv,nv,nv,nv,nv,nv,nv,nv,nv,nv,nv,0,nv,0,0,,,
90,59.97745,30.36554,5.0995,roadside,roadside,roadside,roadside,TU,TU,AT,AT,6,01,31,20,2,2,2,2,Present,2/0,15,0,15,0,18,0,18,0,0,nv,1,nv,nv,nv,nv,nv,nv,nv,nv,nv,nv,nv,0,nv,0,0,,,
91,59.97780,30.36638,5.1580,roadside,roadside,roadside,roadside,U,U,ATU,.,14,41,46,54,M,1,2,1,Present,2/0,3,0,5,0,12,0,12,0,1,nv,1,nv,nv,nv,nv,nv,nv,nv,nv,nv,nv,1,nv,nv,0,,,
92,59.96732,30.38613,5.0505,public_garden,public_garden,public_garden,public_garden,U,U,U,TU,49,89,25,41,M,1,2,2,Present,2/0,18,0,18,0,14,0,14,0,1,nv,1,nv,nv,nv,nv,nv,nv,nv,nv,nv,nv,1,nv,0,1,,,
93,59.94420,30.28647,1.7466,enclosed_courtyard,enclosed_courtyard,enclosed_courtyard,enclosed_courtyard,AT,ATU,ATU,ATU,11,96,11,86,1,3,3,3,Present,0/1,15,0,13,5,15,0,15,0,0,nv,1,1,1,2,1,0,1,na,nv,nv,nv,nv,na,nv,nv,1,,,
94,59.94220,30.28777,1.6167,open_courtyard,open_courtyard,open_courtyard,open_courtyard,T,AT,AT,ATU,34,30,31,25,S,M,3,3,Present,0/3,12,0,12,0,12,0,12,0,0,nv,2,2,2,1,1,1,1,0,1,1,1,1,0,nv,nv,2,,,
95,59.94177,30.28808,1.5902,open_courtyard,open_courtyard,open_courtyard,open_courtyard,T,AT,T,ATU,32,99,35,96,S,2,2,2,Present,1/0,7,0,9,0,9,0,9,0,0,nv,2,1,2,2,1,0,1,0,0,2,1,1,1,nv,nv,0,,,
96,59.93956,30.28389,1.8002,roadside,roadside,roadside,roadside,TU,AT,AT,AT,50,45,17,91,M,1,1,1,Present,1/0,8,0,10,0,10,0,10,0,1,nv,1,nv,nv,nv,nv,nv,nv,nv,nv,nv,nv,nv,0,nv,nv,0,,,
97,59.93955,30.28138,1.9416,enclosed_courtyard,enclosed_courtyard,enclosed_courtyard,enclosed_courtyard,T,T,TU,AT,31,12,20,53,1,1,1,7,Present,7/0,15,0,15,0,15,0,10,5,0,nv,1,nv,nv,nv,nv,nv,nv,nv,nv,nv,nv,0,nv,nv,0,,,
98,59.93797,30.27954,2.0491,roadside,roadside,roadside,roadside,AT,AT,AT,AT,20,61,10,23,1,1,1,0,Lost,.,18,0,18,0,18,0,.,0,nv,1,nv,nv,nv,nv,nv,nv,nv,nv,nv,nv,0,nv,nv,nv,,,
99,59.93774,30.27885,2.0897,roadside,roadside,roadside,roadside,TU,TU,TU,ATU,49,08,25,81,M,6,5,4,Present,0/4,15,0,12,0,15,0,12,0,1,nv,1,2,2,2,2,0,2,0,1,2,2,2,1,nv,nv,1,,,
100,59.936577,30.274873,2.3256,roadside,roadside,roadside,roadside,U,AU,AU,AU,49,52,24,72,S,2,1,0,Lost,.,15,0,15,0,15,0,.,0,nv,na,2,2,1,1,0,1,0,1,1,1,1,0,nv,nv,nv,,,
101,59.933588,30.266115,2.8691,open_courtyard,open_courtyard,open_courtyard,open_courtyard,AST,AST,AST,AST,36,87,22,79,S,0,0,0,Lost,.,10,0,.,.,.,0,nv,nv,nv,nv,nv,nv,nv,nv,nv,nv,nv,nv,nv,nv,nv,nv,nv,,,
102,59.932806,30.262458,3.0894,enclosed_courtyard,enclosed_courtyard,enclosed_courtyard,enclosed_courtyard,TU,TU,TU,TU,23,80,1,09,L,0,0,0,Lost,.,15,0,.,.,.,1,nv,nv,nv,nv,nv,nv,nv,nv,nv,nv,nv,nv,nv,nv,nv,nv,nv,,,
103,59.926542,30.255682,3.6688,roadside,roadside,roadside,roadside,U,U,U,U,8,27,21,98,1,0,0,0,Lost,.,8,0,.,.,.,0,nv,nv,nv,nv,nv,nv,nv,nv,nv,nv,nv,nv,nv,nv,nv,nv,nv,,,
104,59.926901,30.238076,4.5887,roadside,roadside,roadside,roadside,U,U,U,TU,16,36,10,98,M,2,2,2,Present,2/0,3,5,3,5,3,5,3,5,1,nv,1,nv,nv,nv,nv,nv,nv,nv,nv,nv,nv,1,nv,nv,0,,,

105,59.925364,30.237696,4.6945,public_garden,public_garden,public_garden,public_garden,U,STU,ASU,ASU,2,76,0,74,L,4,4,0,Lost,.,16,5,16,5,
16,5,.,1,nv,0,1,2,1,1,0,1,0,1,1,1,1,1,nv,nv,nv,,,
106,59.937207,30.232479,4.7012,open_courtyard,open_courtyard,open_courtyard,open_courtyard,U,TU,TU,ATU,31,88,36,47,S,13,L,L,Present,
4/0,15,0,17,0,15,0,15,0,1,nv,1,1,2,2,2,0,2,0,1,2,1,1,0,nv,nv,1,,,
107,59.954942,30.235179,4.8716,roadside,roadside,roadside,roadside,U,ATU,ATU,ATU,3,30,7,87,L,L,L,L,Present,7/2,5,0,8,0,8,0,8,0,1,nv,1,nv,n
v,nv,nv,nv,nv,nv,nv,nv,nv,nv,1,nv,nv,1,,,
108,59.95472,30.2382,4.7041,public_garden,public_garden,public_garden,public_garden,U,U,U,TU,2,24,41,86,L,L,L,L,Present,3/7,5,0,7,0,7,0,7,
0,1,nv,1,nv,nv,nv,nv,nv,nv,nv,nv,nv,nv,nv,0,nv,nv,0,,,
109,59.94267,30.25070,3.6903,park,park,park,park,U,U,.,TU,100,00,93,79,L,L,.,4,Present,3/1,20,0,20,0,.,20,0,nv,nv,nv,nv,nv,nv,nv,nv,nv,nv,nv,n
v,nv,nv,nv,nv,nv,0,,,
110,59.946086,30.269790,2.7060,enclosed_courtyard,enclosed_courtyard,enclosed_courtyard,enclosed_courtyard,AT,AT,AT,AT,47,41,13,03,1,
1,1,0,Lost,.,12,0,16,0,12,0,.,0,nv,0,0,0,0,nv,nv,nv,nv,nv,nv,nv,nv,0,nv,nv,nv,,,
111,59.94621,30.2735,2.5106,public_garden,public_garden,public_garden,public_garden,U,TU,SU,ASTU,92,70,37,75,L,L,L,L,Present,5/5,15,0,1
1,0,15,0,15,0,na,nv,2,1,2,2,2,0,2,0,1,na,1,2,1,nv,nv,0,,,
112,59.97376,30.24468,5.5587,park,park,park,park,U,U,.,TU,43,08,85,48,L,L,.,12,Present,7/5,20,0,.,.,20,0,1,nv,nv,nv,nv,nv,nv,nv,nv,nv,nv,nv,
nv,nv,nv,nv,1,,,
113,59.98017,30.25562,5.6871,park,park,park,park,U,U,.,TU,57,65,53,11,L,L,.,25+,Present,25/0,20,0,.,.,20,0,1,nv,nv,nv,nv,nv,nv,nv,nv,nv,nv,
nv,nv,nv,nv,nv,2,,,
114,59.930085,30.320314,1.0421,enclosed_courtyard,enclosed_courtyard,enclosed_courtyard,enclosed_courtyard,A,A,A,.,3,58,3,33,1,1,1,.,Miss
ing,.,8,0,8,0,12,0,.,0,0,na,na,na,na,na,0,na,na,na,na,na,na,0,0,nv,nv,,,
115,59.929251,30.319898,1.1274,enclosed_courtyard,enclosed_courtyard,enclosed_courtyard,enclosed_courtyard,U,TU,T,U,19,72,16,14,1,1,2,0
,Lost,.,15,0,15,0,18,0,.,1,0,1,1,2,2,1,0,0,0,na,1,na,na,0,0,nv,nv,,,
116,59.929838,30.318986,1.0536,enclosed_courtyard,enclosed_courtyard,enclosed_courtyard,enclosed_courtyard,U,AT,U,T,9,53,9,63,M,M,M,3,
Present,3/0,15,0,15,0,15,0,15,0,1,0,1,1,2,2,1,0,0,0,0,1,na,na,nv,nv,nv,0,,,
117,59.93628,30.3081,0.5414,public_garden,public_garden,public_garden,public_garden,U,U,U,TU,99,86,67,91,L,L,L,3,Present,3/0,17,5,17,5,17
,5,17,5,1,nv,1,1,na,2,2,0,1,0,1,0,1,0,1,nv,nv,0,,,
118,59.932777,30.294167,1.4128,public_garden,public_garden,public_garden,public_garden,U,U,U,.,54,02,55,87,S,S,S,+,Missing,.,10,0,10,0,10,
0,.,1,nv,1,na,2,2,2,na,0,0,0,2,1,2,0,nv,nv,nv,,,
119,59.937435,30.288729,1.5392,roadside,roadside,roadside,roadside,TU,AU,TU,TU,9,67,11,71,1,1,1,0,Lost,.,6,0,16,0,12,0,.,na,nv,0,1,1,1,1,0,0
,0,0,0,0,0,0,nv,nv,nv,,,
120,59.941070,30.284534,1.7761,roadside,roadside,roadside,roadside,A,A,A,A,29,88,8,13,1,0,0,0,Lost,.,2,0,.,.,.,.,0,nv,nv,nv,nv,nv,nv,nv,nv,nv,
nv,nv,nv,nv,nv,nv,nv,,,
121,59.94275,30.28168,1.9651,open_courtyard,open_courtyard,open_courtyard,open_courtyard,U,U,U,TU,25,94,18,65,M,3,3,3,Present,2/1,10,0
,10,0,13,0,13,0,na,nv,2,nv,nv,nv,nv,nv,nv,nv,nv,nv,nv,nv,0,nv,nv,0,,,
122,59.85087,30.26579,10.2256,open_courtyard,open_courtyard,open_courtyard,open_courtyard,TU,TU,ATU,ATU,.,.,.,M,M,M,M,Present,8/2,6,5,
6,5,13,0,13,0,1,nv,1,nv,nv,nv,nv,nv,nv,nv,nv,nv,nv,nv,1,nv,0,1,,,
123,59.84197,30.33137,10.8553,roadside,roadside,roadside,roadside,TU,TU,ATU,TU,.,.,.,M,M,2,2,Present,2/0,12,0,16,0,18,0,18,0,1,nv,1,nv,nv,nv
,nv,nv,nv,nv,nv,nv,nv,nv,0,nv,1,0,,,
124,59.84119,30.33153,10.9426,open_courtyard,open_courtyard,open_courtyard,open_courtyard,TU,TU,TU,TU,.,.,.,M,L,7+,7,Present,7/0,10,0,13,
0,16,5,16,5,1,nv,2,nv,nv,nv,nv,nv,nv,nv,nv,nv,nv,nv,1,nv,0,0,,,
125,59.83847,30.33631,11.2688,open_courtyard,open_courtyard,open_courtyard,open_courtyard,TU,TU,TU,TU,.,.,.,S,1,1,1,Present,0/1,12,0,16,0
,13,0,13,0,0,nv,2,nv,nv,nv,nv,nv,nv,nv,nv,nv,nv,nv,1,nv,0,0,,,
126,59.83506,30.33601,11.6448,roadside,roadside,roadside,roadside,U,U,U,TU,.,.,.,L,L,L,25+,Present,9/1,17,5,17,5,17,5,20,0,2,nv,2,nv,nv,nv,nv,
nv,nv,nv,nv,nv,nv,nv,nv,nv,0,0,,,
127,59.83169,30.34363,12.0664,public_garden,public_garden,public_garden,public_garden,U,U,U,TU,.,.,.,L,1,0,0,Lost,.,10,0,15,0,.,.,.,na,nv,1,nv,nv
,nv,nv,nv,nv,nv,nv,nv,nv,nv,nv,nv,nv,nv,,,
128,59.83452,30.35256,11.8316,public_garden,public_garden,public_garden,public_garden,U,LU,LU,LTU,.,.,.,M,M,0,6,Present,6/0,15,0,15,0,.,15,
0,1,nv,2,nv,nv,nv,nv,nv,nv,nv,nv,nv,nv,nv,nv,nv,nv,0,,,
129,59.84449,30.41640,11.9659,wasteland,wasteland,roadside_lawn,roadside_lawn,U,.,.,.,U,.,.,.,L,.,0,0,Lost,.,3,5,.,.,.,.,0,nv,nv,nv,nv,nv,nv,nv,nv,nv,
nv,nv,nv,nv,nv,nv,nv,nv,,,
130,59.84489,30.41816,11.9741,wasteland,wasteland,roadside_lawn,roadside_lawn,U,U,U,U,.,.,.,L,L,0,0,Lost,.,6,0,6,0,.,.,.,2,nv,2,nv,nv,nv,nv,nv,nv,
nv,nv,nv,nv,nv,nv,nv,nv,nv,,,
131,59.84819,30.43793,12.2428,park,park,park,park,U,U,.,U,.,.,.,L,L,.,10+,Present,1/9,15,0,15,0,.,7,0,2,nv,nv,nv,nv,nv,nv,nv,nv,nv,nv,nv,nv,nv,
nv,nv,2,,,
132,59.84889,30.46028,12.9315,wasteland,wasteland,asphalted_area,asphalted_area,U,.,A,AU,.,.,.,L,.,0,0,Destroyed,.,13,0,.,.,.,13,0,na,nv,nv,nv,nv
,nv,nv,nv,nv,nv,nv,nv,nv,nv,nv,nv,nv,nv,,,
133,59.850691,30.467267,13.0306,open_courtyard,open_courtyard,open_courtyard,open_courtyard,U,.,U,LTU,.,.,.,L,.,0,0,Lost,.,11,5,.,.,.,.,na,nv,nv,
nv,nv,nv,nv,nv,nv,nv,nv,nv,nv,nv,nv,nv,nv,nv,nv,,,
134,59.85417,30.47264,12.9451,open_courtyard,open_courtyard,open_courtyard,open_courtyard,U,U,TU,ALTU,.,.,.,S,S,1,2,Present,2/0,15,0,15,0
,15,0,18,0,0,nv,2,nv,nv,nv,nv,nv,nv,nv,nv,nv,nv,nv,0,nv,nv,0,,,
135,59.85300,30.47454,13.1133,open_courtyard,open_courtyard,open_courtyard,open_courtyard,U,U,AU,AU,.,.,.,1,1,1,1,Present,0/1,5,0,8,0,8,0,8
,0,0,nv,2,nv,nv,nv,nv,nv,nv,nv,nv,nv,nv,nv,0,nv,nv,0,,,
136,59.85397,30.47838,13.1839,roadside,roadside,road,road,AT,AT,.,.,.,.,.,.,1,1,.,.,.,Destroyed,.,15,0,15,0,.,.,.,1,nv,1,nv,nv,nv,nv,nv,nv,nv,nv,nv,nv,nv,
nv,nv,nv,nv,,,
137,59.85402,30.48114,13.2884,roadside,roadside,road,road,U,U,.,.,.,.,.,.,2,2,.,.,.,Destroyed,.,10,0,10,0,.,.,.,0,nv,1,nv,nv,nv,nv,nv,nv,nv,nv,nv,nv,nv,nv
,nv,nv,nv,,,
138,59.875294,30.439866,9.9694,open_courtyard,open_courtyard,open_courtyard,open_courtyard,U,U,ATU,AT,.,.,.,2,2,2,2,Present,0/2,15,0,15,0,
17,5,19,0,1,nv,2,nv,nv,nv,nv,nv,nv,nv,nv,nv,nv,1,nv,nv,0,,,
139,59.87317,30.43496,9.9509,open_courtyard,open_courtyard,open_courtyard,open_courtyard,AT,AT,ALU,AST,.,.,.,1,1,25+,25+,Present,7/3,15,
0,15,0,11,0,18,0,0,nv,nv,nv,nv,nv,nv,nv,nv,nv,nv,nv,nv,nv,1,nv,nv,2,,,
140,59.87356,30.43364,9.8688,open_courtyard,open_courtyard,open_courtyard,open_courtyard,TU,TU,TU,AT,.,.,.,1,0,0,0,Lost,.,15,0,.,.,.,.,1,nv,nv,
nv,nv,nv,nv,nv,nv,nv,nv,nv,nv,nv,nv,nv,nv,nv,nv,,,
141,59.89362,30.41493,7.5418,open_courtyard,open_courtyard,open_courtyard,open_courtyard,TU,TU,AU,AUT,.,.,.,3,1,1,1,Present,2/0,10,0,15,0
,20,0,20,0,1,nv,1,nv,nv,nv,nv,nv,nv,nv,nv,nv,nv,nv,1,nv,nv,0,,,
142,59.89563,30.41772,7.5127,open_courtyard,open_courtyard,open_courtyard,open_courtyard,TU,ATU,ALU,AT,.,.,.,M,L,10+,10,Present,3/7,8,0,
8,0,16,5,16,5,1,nv,2,nv,nv,nv,nv,nv,nv,nv,nv,nv,nv,nv,0,nv,nv,0,,,
143,59.90497,30.41892,6.9443,public_garden,public_garden,public_garden,public_garden,AT,AT,AU,AU,27,33,40,37,2,1,5,4,Present,0/4,15,0,1
5,0,13,5,13,5,0,nv,2,nv,nv,nv,nv,nv,nv,nv,nv,nv,nv,nv,1,nv,nv,1,,,
144,59.907778,30.417778,6.7230,public_garden,public_garden,public_garden,public_garden,U,U,AU,AT,33,36,70,98,M,M,5+,5,Present,8/1,7,5,8
,5,13,5,13,5,2,nv,2,nv,nv,nv,nv,nv,nv,nv,nv,nv,nv,nv,nv,nv,nv,0,,,
145,59.907818,30.393772,5.6089,open_courtyard,open_courtyard,open_courtyard,open_courtyard,U,U,U,.,11,75,59,72,M,L,L,.,Missing,.,15,0,11
,5,14,0,.,1,nv,1,nv,nv,nv,nv,nv,nv,nv,nv,nv,nv,nv,1,nv,nv,nv,,,
146,59.90947,30.40247,5.8953,roadside,roadside,roadside,roadside,TU,TU,ATU,ATU,8,03,13,02,2,2,2,2,Present,2/0,15,0,20,0,15,0,15,0,0,nv,2,
nv,nv,nv,nv,nv,nv,nv,nv,nv,nv,nv,0,nv,nv,0,,,
147,59.907833,30.404017,6.0705,roadside,roadside,roadside,roadside,A,A,A,A,5,06,5,82,1,0,0,0,Lost,.,15,0,.,.,.,.,0,nv,nv,nv,nv,nv,nv,nv,nv,nv,
nv,nv,nv,nv,nv,nv,nv,,,

148,59.916366,30.399434,5.35045,roadside,roadside,roadside,roadside,TU,STU,ATU,TU,19,14,4,69,M,M,6,0,Lost,.,12,0,16,5,6,0,.,2,nv,1,nv,nv,
nv,nv,nv,nv,nv,nv,nv,nv,nv,0,nv,nv,nv,,,
149,59.91795,30.40123,5.35973,public_garden,public_garden,public_garden,public_garden,U,U,U,U,72,62,57,95,L,L,6,6,Present,0/6,15,0,15,0,2
0,0,20,0,2,nv,2,nv,nv,nv,nv,nv,nv,nv,nv,nv,nv,nv,1,nv,nv,1,,,
150,59.943056,30.371389,3.15982,public_garden,public_garden,public_garden,public_garden,U,U,U,AU,54,65,57,81,L,2,S,2,Present,0/2,15,0,1
5,0,15,0,20,0,1,nv,2,2,2,2,2,0,2,0,1,1,1,2,0,nv,nv,0

**Data S2**. Characteristics of grid cells.

Column 1: Row number (from 1, northernmost, to 21, southernmost).
Column 2: Column number (from 1, westernmost, to 5, easternmost).
Column 3: Cell status in 1986.
Column 4: Cell status in 2000.
Column 5: Cell status in 2001.
Column 6: Cell status in 2006.
Column 7: Cell status in 2007.
Column 8: Cell status in 2008.
Column 9: Cell status in 2009.
Column 10: Cell status in 2010.
Column 11: Cell status in 2011.
Column 12: Cell status in 2012.
Column 13: Cell status in 2025.

1,3,unsuitable,occupied,unsuitable,unsuitable,vacant,vacant,occupied,occupied,vacant,vacant,unsuitable
1,4,unsuitable,occupied,occupied,not_visited,vacant,occupied,occupied,occupied,occupied,occupied,vacant
1,5,occupied,vacant,occupied,not_visited,occupied,occupied,occupied,occupied,occupied,vacant,vacant
2,2,unsuitable,vacant,occupied,unsuitable,vacant,occupied,occupied,occupied,vacant,occupied,vacant
2,3,unsuitable,occupied,occupied,not_visited,vacant,vacant,occupied,occupied,occupied,vacant,vacant
2,4,unsuitable,occupied,occupied,not_visited,vacant,vacant,occupied,occupied,occupied,occupied,vacant
2,5,occupied,unsuitable,unsuitable,not_visited,vacant,vacant,vacant,vacant,vacant,occupied,unsuitable
3,1,unsuitable,occupied,occupied,unsuitable,unsuitable,unsuitable,unsuitable,unsuitable,unsuitable,unsuitable,unsuitable
3,2,unsuitable,occupied,occupied,not_visited,occupied,occupied,occupied,occupied,occupied,occupied,vacant
3,3,unsuitable,vacant,occupied,not_visited,vacant,vacant,vacant,vacant,vacant,vacant,not_assessed
3,4,occupied,vacant,unsuitable,not_visited,vacant,vacant,occupied,occupied,occupied,occupied,occupied
3,5,unsuitable,unsuitable,unsuitable,not_visited,vacant,not_assessed,not_assessed,not_assessed,not_assessed,not_assessed,not_assessed
4,1,vacant,occupied,occupied,not_visited,vacant,occupied,occupied,occupied,occupied,occupied,occupied
4,2,unsuitable,occupied,occupied,not_visited,occupied,occupied,occupied,occupied,occupied,occupied,occupied
4,3,occupied,vacant,occupied,not_visited,vacant,occupied,occupied,occupied,occupied,vacant,occupied
4,4,occupied,occupied,occupied,not_visited,occupied,vacant,occupied,occupied,occupied,occupied,unsuitable
4,5,occupied,vacant,vacant,not_visited,unsuitable,unsuitable,unsuitable,unsuitable,unsuitable,unsuitable,unsuitable
5,1,occupied,occupied,occupied,not_visited,vacant,occupied,occupied,occupied,occupied,occupied,occupied
5,2,occupied,occupied,occupied,not_visited,occupied,occupied,occupied,occupied,occupied,occupied,occupied
5,3,occupied,vacant,occupied,not_visited,vacant,vacant,occupied,occupied,occupied,occupied,occupied
5,4,unsuitable,vacant,unsuitable,not_visited,vacant,vacant,occupied,vacant,vacant,occupied,occupied
5,5,unsuitable,occupied,not_assessed,not_visited,vacant,vacant,occupied,occupied,occupied,occupied,not_assessed
6,1,unsuitable,not_assessed,unsuitable,not_visited,vacant,occupied,occupied,occupied,occupied,occupied,not_assessed
6,2,unsuitable,vacant,occupied,not_visited,vacant,occupied,occupied,occupied,occupied,occupied,occupied
6,3,unsuitable,occupied,occupied,not_visited,vacant,occupied,occupied,occupied,occupied,occupied,occupied
6,4,vacant,unsuitable,unsuitable,unsuitable,vacant,vacant,vacant,vacant,vacant,vacant,unsuitable
6,5,unsuitable,unsuitable,not_assessed,not_visited,vacant,occupied,occupied,occupied,occupied,vacant,unsuitable
7,1,unsuitable,unsuitable,unsuitable,unsuitable,unsuitable,unsuitable,unsuitable,unsuitable,unsuitable,unsuitable,unsuitable
7,2,unsuitable,unsuitable,unsuitable,not_visited,vacant,occupied,occupied,occupied,occupied,occupied,vacant
7,3,occupied,unsuitable,unsuitable,not_visited,vacant,not_assessed,unsuitable,unsuitable,unsuitable,unsuitable,unsuitable
7,4,unsuitable,occupied,occupied,unsuitable,unsuitable,unsuitable,unsuitable,unsuitable,unsuitable,unsuitable,unsuitable
7,5,unsuitable,unsuitable,unsuitable,not_visited,unsuitable,unsuitable,unsuitable,unsuitable,unsuitable,unsuitable,unsuitable
8,1,unsuitable,occupied,occupied,unsuitable,unsuitable,unsuitable,unsuitable,unsuitable,unsuitable,unsuitable,unsuitable
8,2,occupied,vacant,unsuitable,unsuitable,unsuitable,unsuitable,unsuitable,unsuitable,unsuitable,unsuitable,unsuitable
8,3,occupied,occupied,occupied,unsuitable,unsuitable,unsuitable,unsuitable,unsuitable,unsuitable,unsuitable,unsuitable
8,4,unsuitable,unsuitable,unsuitable,not_visited,vacant,occupied,occupied,occupied,occupied,occupied,occupied
8,5,unsuitable,unsuitable,unsuitable,unsuitable,vacant,vacant,occupied,occupied,occupied,vacant,unsuitable
9,1,unsuitable,occupied,unsuitable,vacant,vacant,occupied,occupied,occupied,occupied,occupied,occupied
9,2,occupied,occupied,occupied,vacant,unsuitable,unsuitable,unsuitable,unsuitable,unsuitable,unsuitable,unsuitable
9,3,unsuitable,occupied,occupied,vacant,vacant,occupied,occupied,occupied,occupied,occupied,occupied
9,4,vacant,occupied,occupied,not_assessed,vacant,vacant,occupied,occupied,occupied,occupied,not_assessed
9,5,occupied,occupied,occupied,unsuitable,unsuitable,unsuitable,unsuitable,unsuitable,unsuitable,unsuitable,unsuitable
10,1,unsuitable,unsuitable,occupied,vacant,vacant,occupied,occupied,occupied,occupied,vacant,occupied
10,2,unsuitable,occupied,occupied,vacant,occupied,occupied,occupied,not_visited,occupied,occupied,occupied
10,3,unsuitable,occupied,occupied,vacant,occupied,occupied,occupied,not_visited,occupied,occupied,occupied
10,4,vacant,unsuitable,occupied,vacant,occupied,vacant,occupied,occupied,occupied,vacant,occupied
10,5,occupied,vacant,unsuitable,vacant,vacant,occupied,occupied,occupied,occupied,occupied,occupied
11,1,unsuitable,unsuitable,unsuitable,not_visited,unsuitable,unsuitable,unsuitable,unsuitable,unsuitable,unsuitable,unsuitable
11,2,unsuitable,occupied,occupied,not_visited,vacant,occupied,occupied,vacant,occupied,occupied,occupied
11,3,unsuitable,vacant,occupied,vacant,vacant,occupied,occupied,not_visited,occupied,occupied,vacant
11,4,unsuitable,occupied,occupied,occupied,vacant,vacant,occupied,not_visited,occupied,vacant,vacant

11,5,unsuitable,occupied,occupied,unsuitable,unsuitable,unsuitable,unsuitable,unsuitable,unsuitable,unsuitable,unsuitable
12,1,occupied,unsuitable,unsuitable,unsuitable,unsuitable,unsuitable,unsuitable,unsuitable,unsuitable,unsuitable,unsuitable
12,2,unsuitable,occupied,occupied,not_visited,unsuitable,unsuitable,unsuitable,unsuitable,unsuitable,unsuitable,unsuitable
12,3,unsuitable,occupied,occupied,occupied,vacant,vacant,occupied,occupied,occupied,occupied,not_assessed
12,4,unsuitable,unsuitable,unsuitable,not_visited,vacant,occupied,occupied,not_visited,occupied,vacant,vacant
12,5,vacant,occupied,occupied,vacant,vacant,occupied,occupied,not_visited,occupied,vacant,occupied
13,1,occupied,occupied,occupied,vacant,vacant,vacant,vacant,occupied,occupied,occupied,vacant
13,2,unsuitable,occupied,occupied,vacant,vacant,occupied,occupied,occupied,occupied,occupied,vacant
13,3,unsuitable,unsuitable,unsuitable,vacant,vacant,vacant,occupied,not_visited,occupied,occupied,occupied
13,4,unsuitable,occupied,occupied,unsuitable,unsuitable,unsuitable,unsuitable,unsuitable,unsuitable,unsuitable,unsuitable
13,5,vacant,vacant,unsuitable,vacant,vacant,occupied,occupied,not_visited,occupied,vacant,vacant
14,1,vacant,unsuitable,unsuitable,vacant,vacant,occupied,occupied,occupied,occupied,occupied,occupied
14,2,unsuitable,occupied,occupied,vacant,vacant,occupied,occupied,occupied,occupied,occupied,occupied
14,3,occupied,unsuitable,not_assessed,unsuitable,unsuitable,unsuitable,unsuitable,unsuitable,unsuitable,unsuitable,unsuitable
14,4,vacant,vacant,occupied,vacant,vacant,vacant,occupied,occupied,occupied,occupied,occupied
14,5,unsuitable,unsuitable,unsuitable,unsuitable,unsuitable,unsuitable,unsuitable,unsuitable,unsuitable,unsuitable,unsuitable
15,1,vacant,unsuitable,not_assessed,unsuitable,unsuitable,unsuitable,unsuitable,unsuitable,unsuitable,unsuitable,unsuitable
15,2,unsuitable,unsuitable,unsuitable,not_visited,unsuitable,unsuitable,unsuitable,unsuitable,unsuitable,unsuitable,unsuitable
15,3,unsuitable,occupied,occupied,not_visited,vacant,occupied,not_assessed,not_assessed,not_assessed,not_assessed,not_assessed
15,4,vacant,occupied,occupied,vacant,vacant,occupied,occupied,occupied,occupied,occupied,vacant
15,5,unsuitable,unsuitable,unsuitable,unsuitable,unsuitable,unsuitable,unsuitable,unsuitable,unsuitable,unsuitable,unsuitable
16,1,unsuitable,unsuitable,unsuitable,vacant,vacant,vacant,occupied,occupied,occupied,occupied,vacant
16,2,unsuitable,occupied,unsuitable,unsuitable,unsuitable,unsuitable,unsuitable,unsuitable,unsuitable,unsuitable,unsuitable
16,3,unsuitable,occupied,occupied,not_visited,unsuitable,unsuitable,unsuitable,unsuitable,unsuitable,unsuitable,unsuitable
16,4,vacant,unsuitable,occupied,vacant,vacant,occupied,occupied,vacant,occupied,vacant,occupied
16,5,unsuitable,occupied,occupied,vacant,vacant,occupied,occupied,occupied,occupied,vacant,occupied
17,1,unsuitable,vacant,occupied,unsuitable,unsuitable,unsuitable,unsuitable,unsuitable,unsuitable,unsuitable,unsuitable
17,2,unsuitable,unsuitable,unsuitable,unsuitable,unsuitable,unsuitable,unsuitable,unsuitable,unsuitable,unsuitable,unsuitable
17,3,unsuitable,unsuitable,occupied,occupied,vacant,occupied,occupied,occupied,occupied,occupied,vacant
17,4,occupied,occupied,occupied,not_visited,not_visited,vacant,occupied,occupied,vacant,vacant,vacant
17,5,unsuitable,occupied,occupied,vacant,vacant,occupied,occupied,occupied,occupied,occupied,unsuitable
18,1,unsuitable,occupied,not_assessed,not_assessed,vacant,occupied,occupied,occupied,vacant,vacant,vacant
18,2,unsuitable,occupied,occupied,unsuitable,unsuitable,unsuitable,unsuitable,unsuitable,unsuitable,unsuitable,unsuitable
18,3,unsuitable,occupied,occupied,not_visited,vacant,occupied,occupied,occupied,occupied,occupied,vacant
18,4,occupied,occupied,unsuitable,vacant,vacant,occupied,vacant,occupied,occupied,occupied,vacant
18,5,unsuitable,occupied,occupied,vacant,vacant,occupied,occupied,occupied,occupied,occupied,occupied
19,1,unsuitable,occupied,occupied,vacant,vacant,occupied,occupied,occupied,occupied,occupied,vacant
19,2,unsuitable,occupied,occupied,vacant,occupied,occupied,occupied,occupied,occupied,vacant,vacant
19,3,unsuitable,occupied,occupied,vacant,vacant,occupied,occupied,occupied,occupied,vacant,unsuitable
19,4,unsuitable,unsuitable,unsuitable,unsuitable,unsuitable,unsuitable,unsuitable,unsuitable,unsuitable,unsuitable,unsuitable
19,5,unsuitable,occupied,vacant,vacant,occupied,occupied,occupied,vacant,occupied,occupied,vacant
20,1,unsuitable,unsuitable,unsuitable,vacant,vacant,occupied,occupied,vacant,occupied,vacant,occupied
20,2,unsuitable,occupied,occupied,not_assessed,vacant,vacant,vacant,vacant,occupied,not_assessed,unsuitable
20,3,unsuitable,unsuitable,occupied,vacant,vacant,occupied,vacant,vacant,occupied,vacant,vacant
20,4,unsuitable,unsuitable,unsuitable,unsuitable,unsuitable,unsuitable,unsuitable,unsuitable,unsuitable,unsuitable,not_assessed
20,5,unsuitable,unsuitable,unsuitable,not_visited,unsuitable,unsuitable,unsuitable,unsuitable,unsuitable,unsuitable,unsuitable
21,1,unsuitable,unsuitable,unsuitable,unsuitable,unsuitable,unsuitable,unsuitable,unsuitable,unsuitable,unsuitable,unsuitable
21,2,unsuitable,unsuitable,unsuitable,not_visited,unsuitable,unsuitable,unsuitable,unsuitable,unsuitable,unsuitable,unsuitable
21,3,unsuitable,unsuitable,unsuitable,not_visited,unsuitable,unsuitable,unsuitable,unsuitable,unsuitable,unsuitable,unsuitable
21,4,occupied,occupied,occupied,unsuitable,unsuitable,unsuitable,unsuitable,unsuitable,unsuitable,unsuitable,unsuitable
21,5,unsuitable,unsuitable,unsuitable,vacant,vacant,occupied,occupied,occupied,occupied,occupied,occupied
